# Causal relationship between the gut microbiome and basal cell carcinoma, melanoma skin cancer, ease of skin tanning: evidence from three two-sample mendelian randomisation studies

**DOI:** 10.3389/fimmu.2024.1279680

**Published:** 2024-01-18

**Authors:** Jiaqi Lou, Shengyong Cui, Jiliang Li, Guoying Jin, Youfen Fan, Neng Huang

**Affiliations:** Burn Department, Ningbo No. 2 Hospital, Ningbo, Zhejiang, China

**Keywords:** gut microbiome, basal cell carcinoma, melanoma skin cancer, ease of skin tanning, Mendelian randomization, MR, GWAS, genetics

## Abstract

**Objectives:**

The present study used publicly available genome-wide association study (GWAS) summary data to perform three two-sample Mendelian randomization (MR) studies, aiming to examine the causal links between gut microbiome and BCC, melanoma skin cancer, ease of skin tanning.

**Methods:**

SNPs associated with exposures to basal cell carcinoma, melanoma skin cancer and ease of skin tanning from the genome-wide association study data of UK Biobank and MRC-IEU (MRC Integrative Epidemiology Unit), and the meta-analysis data from Biobank and MRC-IEU were used as instrumental variables (IVs). The casual estimates were assessed with a two-sample Mendelian randomisation test using the inverse-variance-weighted (IVW) method, Wald ratio, MR-Egger method, maximum likelihood, weighted median, simple mode, and weighted mode.

**Results:**

After the application of MR analysis, diffirent effects of multiple groups of gut microbiota was observed for BCC, melanoma skin cancer and ease of skin tanning. The relationships between the gut microbiome and BCC, melanoma skin cancer, ease of skin tanning were supported by a suite of sensitivity analyses, with no statistical evidence of instrument heterogeneity or horizontal pleiotropy. Further investigation is required to explore the relationship between between the gut microbiome and BCC, melanoma skin cancer, ease of skin tanning.

**Conclusion:**

Our study initially identified potential causal roles between the gut microbiome and BCC, melanoma skin cancer, ease of skin tanning, and highlighted the role of gut microbiome in the progression of basal cell carcinoma, melanoma skin cancer, ease of skin tanning.

## Background

The gut microbiota is a complex community of microbial balance that varies significantly between individuals. It undergoes constant changes throughout individual development and maintains a certain homeostasis in adulthood (1). The intestinal flora affects not only the absorption of nutrients but also the permeability of the intestinal mucosa, intestinal immune cells, and the secretion of various proteins regulated by the intestine (2, 3). This imbalance of intestinal flora can lead to related diseases. Numerous microbiological studies have gradually confirmed the relationship between intestinal flora imbalance and the occurrence of type-2 diabetes (4), obesity (5), fatty liver (6), atherosclerosis (7), cancer (8), and other chronic diseases (9).

The skin covers the surface of the human body, directly contacting the external environment, and providing protection against various external factors. In recent years, researchers have shown a growing interest in comprehending the role of the human microbiome in skin diseases. Several studies have confirmed the relationship between intestinal flora imbalance and inflammatory skin diseases, including psoriasis (10), acne (11), seborrheic dermatitis (12) and alopecia areata (13).

Basal cell carcinoma (BCC) is one of the most common low-grade malignant cutaneous tumors. Epidemiological studies have identified a relationship between BCC and factors such as family history (14), ultraviolet radiation (15), exposure to harmful chemicals, ionizing radiation (16), and immunosuppressive therapy (17). Prolonged exposure to ultraviolet radiation leads to the secretion of various inflammatory cytokines by skin cells, which contribute to the development of skin erythema, photoaging, immunosuppression, and ultimately, skin cancer (18). However, there is currently no study that has explored the potential relationship between BCC and gut microbiota.

Melanoma, a malignant skin tumor, primarily arises from the malignant transformation of skin melanocytes. It is characterized by its high incidence, high malignancy, propensity for metastasis, and high mortality rate (19). Immune checkpoint inhibition therapy is a widely employed treatment for melanoma (20). Preliminary studies in both mice (21) and humans (22) have indicated a potential impact of intestinal flora on immune checkpoint therapy for melanoma. Furthermore, several studies (23–25) have demonstrated that supplementing with probiotics can influence the effectiveness of tumor immunotherapy through the modulation of intestinal flora. However, the precise underlying mechanism remains incompletely understood. The specific bacterial characteristics that can contribute to a clinical benefit are currently unidentified.

The tanning response is determined by an increase in melanin production in melanocytes stimulated by ultraviolet radiation (26). A recent study revealed a genetic correlation between the propensity for skin tanning and the risk of non-melanoma skin cancer (27). Additionally, studies have indicated that certain individuals who exhibit a tanning response to sunlight exposure may have an increased risk of developing skin diseases. However, the research exploring the potential impact of gut microbiota on the relationship between skin tanning and skin cancer is currently lacking (28).

Numerous genome-wide association studies (GWASs) have examined the associations between genetic variations and diseases or phenotypes (29). Mendelian randomization is a robust statistical method for evaluating causation. It employs genetic variations significantly associated with exposure as instrumental variables to assess the causal relationship between the exposure and the outcome (30). Two-sample Mendelian randomization (MR) analysis can provide causal estimates by utilizing SNP-exposure and SNP-outcome associations derived from independent GWAS studies (31). This study utilizes GWAS and MR analysis to examine the causal links between gut microbiome and BCC, melanoma skin cancer, ease of skin tanning.

## Methods

**Figure 1:**
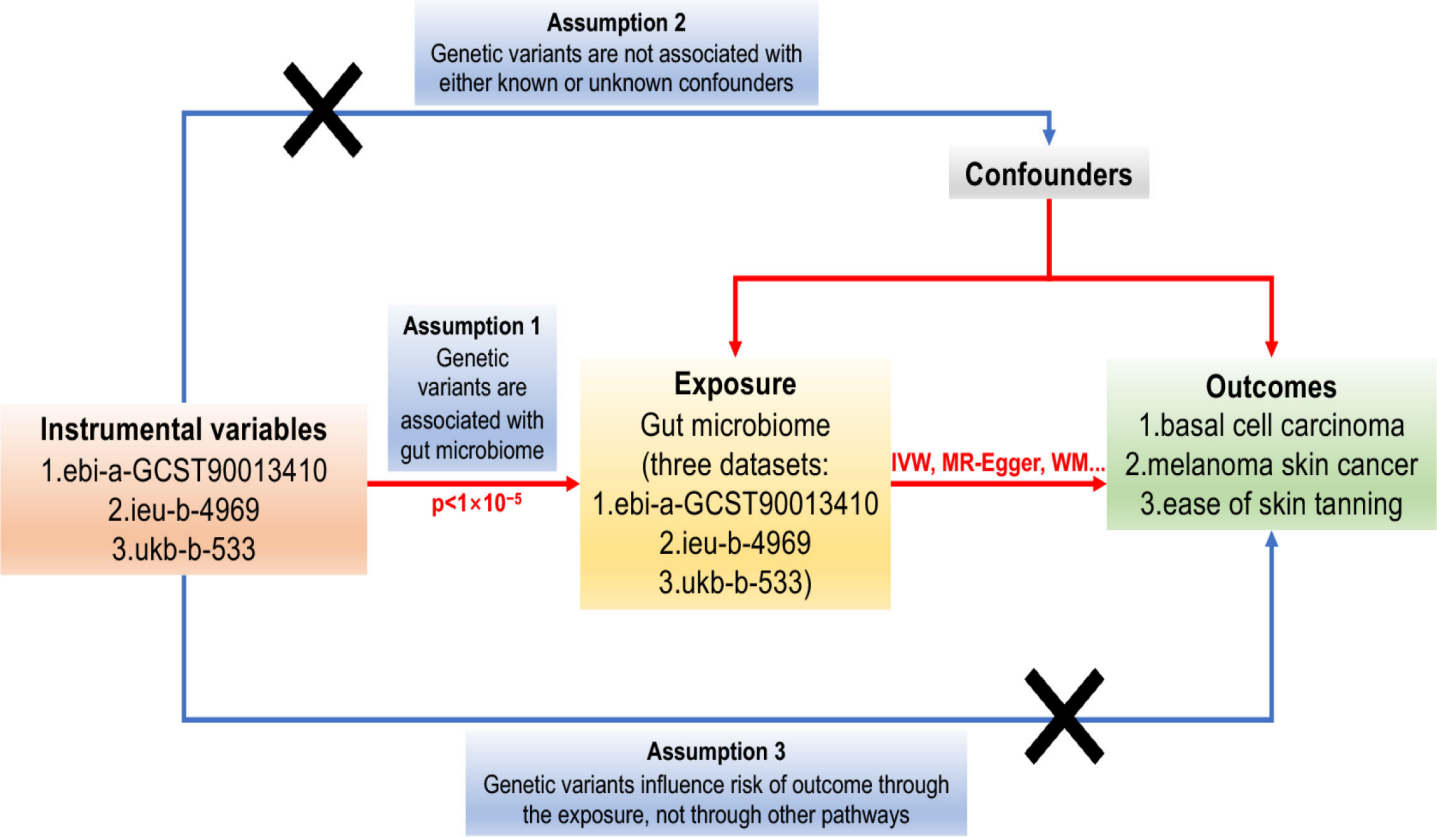
The Directed Acyclic Graph (DAG) representing the Mendelian Randomisation (MR) framework employed to investigate the causal relationship between the gut microbiome and basal cell carcinoma, melanoma skin cancer, and ease of skin tanning. The MR analysis is guided by three crucial instrumental variable assumptions: (1) The instrumental variables must exhibit a strong association with the gut microbiome (p<1×10^-5^). (2) The instrumental variables must not be associated with any potential confounders that could influence the relationship between the gut microbiome and basal cell carcinoma, melanoma skin cancer, and ease of skin tanning. (3) The instrumental variables should solely impact the risk of basal cell carcinoma, melanoma skin cancer, and ease of skin tanning through their influence on the gut microbiome. The instrumental variables are represented by single-nucleotide polymorphisms (SNPs), and the MR analysis employs the Inverse Variance Weighted (IVW) and Weighted Median (WM) methods to estimate causal relationships.

Mendelian Randomisation analysis relies on three critical assumptions (32): (1) The IVs used in the analysis are strongly associated with the exposure of interest; (2) The IVs are not related to confounding factors that may influence the relationship between exposure and outcome; (3) The IVs solely affect the outcome through their influence on the exposure. These assumptions are illustrated in **Figure 1**.

Given that our study utilized data from published studies and publicly available databases, there was no need to seek additional ethical approval from an institutional review board.

### Mendelian randomization

#### Study design

The summary-level data used in the two-sample mendelian randomisation analysis was sourced from the IEU Open GWAS database (https://gwas.mrcieu.ac.uk/) (33) and UK Biobank (https://www.ukbiobank.ac.uk) (34). The data comprised three datasets, each corresponding to different traits: (1) Basal cell carcinoma trait, with GWAS ID: ebi-a-GCST90013410, included 392,871 individuals of European descent from the UK Biobank in 2021. (2) Melanoma skin cancer trait, with GWAS ID: ieu-b-4969, consisted of 375,767 individuals of European descent from the UK Biobank in 2021. (3) Ease of skin tanning trait, with GWAS ID: ukb-b-533, encompassed 453,065 individuals of European descent from the MRC-IEU in 2018.

All the traits related to the gut microbiome were also derived from these three datasets. It is important to note that the initial GWAS studies were conducted with authorization from the relevant ethics committee, and all participants provided informed consent for their data to be used in research.

#### Assumptions of Mendelian randomization study

In this mendelian randomisation research, three fundamental assumptions must be satisfied to establish causal relationships: (1) The GIVs used in the analysis must demonstrate a significant relationship with the exposure of interest. (2) The GIVs must not be associated with any potential confounding factors that could bias the relationship between exposure and outcome. (3) The GIVs should only influence the risk of the outcome through their effect on the exposure. The assumptions and design of the MR study are depicted in **Figure 1**, which illustrates the causal relationships between the genetic instrument variables, exposures, and outcomes.

#### Exposure data

IVs used in this study were selected from a GWAS dataset of the Medical Research Center-Integrative Epidemiology Unit (MRC-IEU) and UK Biobank GWAS Pipeline. These IVs are SNPs that are known to be associated with the composition of the human gut microbiome. The study aims to investigate the potential causal relationship between autosomal human genetic variation and gut microbial communities.

#### Outcome data

The GWAS summary statistics data for basal cell carcinoma of European ancestry (GWAS ID: ebi-a-GCST90013410, including 17,416 cases and 375,455 controls), melanoma skin cancer of European ancestry (GWAS ID: ieu-b-4969, including 3751 cases and 372,016 controls), and ease of skin tanning of European ancestry (GWAS ID: ukb-b-533, cases and controls were not reported numerically) were obtained from the IEU Open GWAS database. After thorough screening of the dataset and excluding duplicate studies and non-European ancestry samples, the GWAS summary-level data relevant to the associations between genetic variants and basal cell carcinoma, melanoma skin cancer, and ease of skin tanning were retained, encompassing data from the UK Biobank and the MRC-IEU. Detailed information on the datasets can be found in **Additional files: Supplementary Tables 1–3**.

#### Instrumental variable

**Figure 2:**
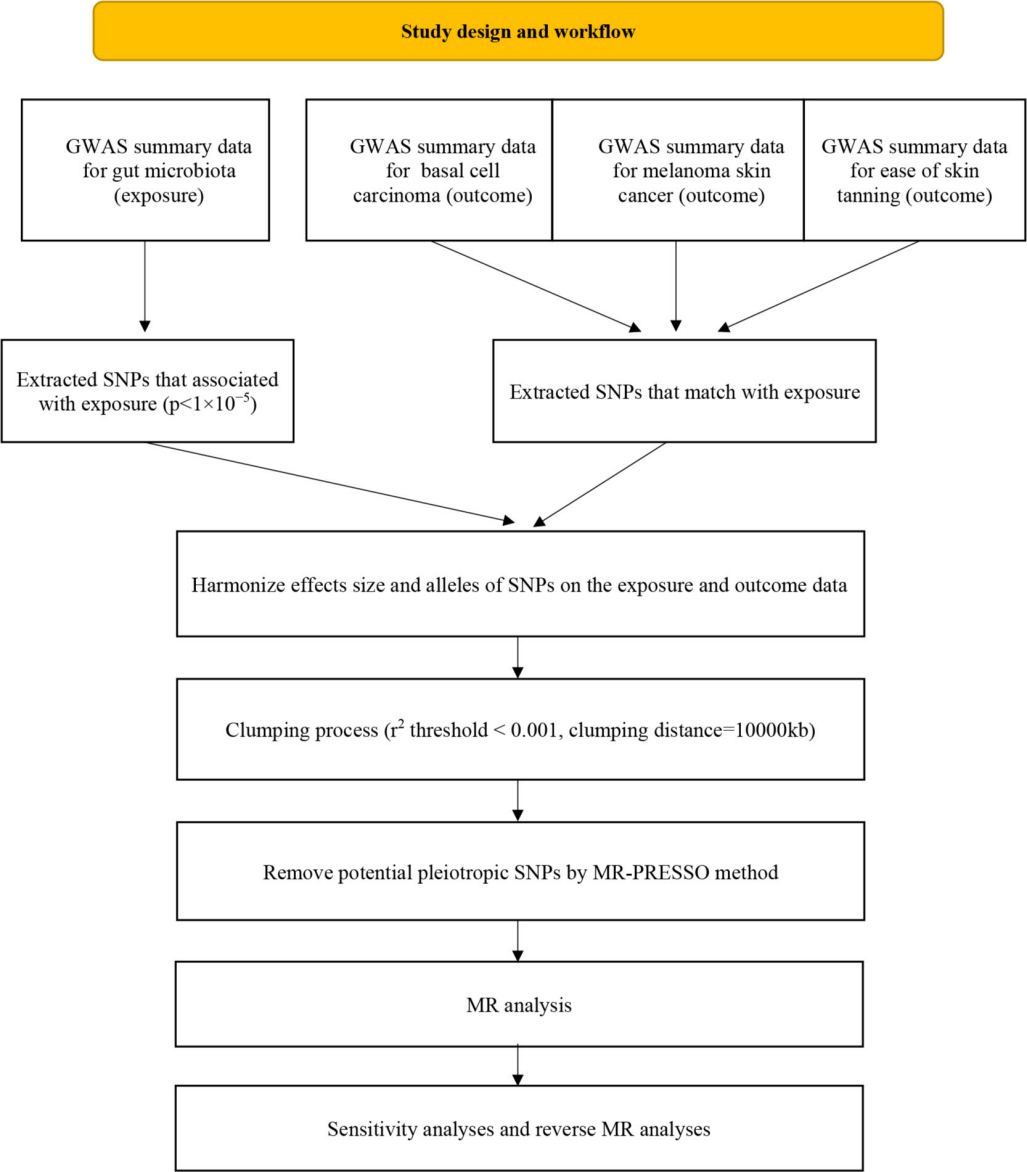
Flow chart of this study.

The flowchart of the study is presented in **Figure 2**. Briefly, the gut microbiota served as the exposure, whereas basal cell carcinoma, melanoma skin cancer and ease of skin tanning served as the outcomes.

To establish a reliable and accurate causal relationship between the gut microbiome and the risk of basal cell carcinoma, melanoma skin cancer, and ease of skin tanning, rigorous quality control measures were employed to select the most suitable IVs. Firstly, SNPs that exhibited significant associations with the gut microbiome were chosen as IVs. Two different thresholds were applied for this selection process. The first threshold involved selecting SNPs with p-values smaller than the genome-wide statistical significance threshold of 5×10^-8^ as IVs. However, this approach yielded only a limited number of gut microbiota as IVs. In order to explore additional associations between basal cell carcinoma, melanoma skin cancer, ease of skin tanning, and gut microbiota for a more comprehensive analysis, a second threshold was used, selecting SNPs with p-values smaller than the genome-wide significance level of 1×10^-5^ as the second set of IVs to uncover more potential causal relationships.

Secondly, a minor allele frequency (MAF) threshold of 0.01 was applied to ensure the reliability of the variant of interest. Thirdly, an essential principle of the MR method is to ensure that there is no linkage disequilibrium (LD) between the included IVs, as strong LD may lead to biased results. To assess LD among the included SNPs in our study, an aggregation process was implemented, with an R^2^ threshold of less than 0.001 and a clustering distance of 10000kb.

Fourthly, in MR, it is crucial to ensure that the effects of SNPs on exposure correspond to the same alleles as the effects on outcomes. To avoid any distortions in strand orientation or allelic coding, palindromic SNPs (e.g., A/T or G/C alleles) were removed from the analysis. During the coordination process, alleles were aligned to the human genome reference sequence, and any ambiguous or duplicate SNPs were removed.

Furthermore, MR-Egger regression tests (35) were conducted to monitor the potential effect of horizontal pleiotropy. Any remaining pleiotropic SNPs were removed from the list of SNPs used in the subsequent MR analysis to maintain the integrity and accuracy of the results.

#### MR analysis

To comprehensively investigate the relationship between the gut microbiome and basal cell carcinoma, melanoma skin cancer, and ease of skin tanning, three separate univariable two-sample MR analyses were conducted for each trait. The primary causal effect estimation method used was the IVW approach (36), which calculated the combined effect of all SNPs included in the study. To ensure the reliability and robustness of the results, multiple additional approaches, including the Wald ratio, MR-Egger, maximum likelihood, weighted median, simple mode, and weighted mode, were employed to examine the data (37, 38).

Given the potential heterogeneity arising from variations in analysis platforms, experimental setups, inclusion populations, and SNPs, it was important to assess heterogeneity in the two-sample MR analysis. The primary IVW and MR-Egger approaches were used for this purpose. If the p-value for the inclusion of instrumental variables exceeded 0.05, it indicated homogeneity, and any effect of heterogeneity on the assessment of causal effects was disregarded. However, in cases where heterogeneity was present, the IVW (multiplicative random effects) approach was utilized to estimate the effect size.

It is crucial in MR analysis to ensure that the fundamental assumptions are met, and pleiotropy can be a potential violation of these assumptions. Pleiotropy occurs when a genetic instrument directly influences the outcome without affecting the exposure of interest. To investigate the presence of pleiotropy, the Egger model’s intercept was used as a statistical assessment; a deviation from zero suggests the presence of directional pleiotropy.

Additionally, to evaluate the robustness of the results, a sensitivity analysis using the leave-one-out approach was performed. This involved conducting the MR analysis again, removing one SNP at a time. If any potentially influential SNPs were identified during the sensitivity analysis, caution was exercised in drawing inferences from the results. These comprehensive analyses aimed to ensure the validity and credibility of the findings in exploring the causal relationships between the gut microbiome and the three skin-related traits.

#### Heterogeneity

A test for heterogeneity was conducted using Cochran’s Q statistics (39) and the two-sample MR package between the IVs. Cochran’s Q statistics assess the variability among the IVs and can provide evidence for heterogeneity and potentially identify invalid instruments. Specifically, if the value of Q is larger than the number of instruments minus one, it indicates the presence of heterogeneity and raises concerns about the validity of the instruments. Additionally, Q statistics with a significant p-value less than 0.05 suggest the presence of heterogeneity among the instruments, further warranting investigation and careful interpretation of the results.

#### Sensitivity

In order to evaluate the stability of the results, an array of sensitivity analyses was executed. A Leave-one-out analysis was initiated to scrutinize whether a single SNP was the driving force behind the causal signal (40). This strategy juxtaposes the variance articulated by the IVs for both the exposure and the outcome. Should the IVs elucidate a more profound variance in exposure than in the outcome, the unveiled causal association can conceivably be considered directionally credible. Moreover, we determined F statistics to examine any potential weak instrument bias. Instruments with an F-value falling below 10 were categorized as weak and subsequently omitted. This statistic helps assess the validity and strength of the instrumental variables used in the Mendelian randomization analysis.

## Results

Based on the GWAS of European ancestry, we identified a total of seven independent SNPs that were associated with the gut microbiome and basal cell carcinoma, four independent SNPs associated with the gut microbiome and melanoma skin cancer, and fourteen independent SNPs associated with the gut microbiome and ease of skin tanning at a genome-wide significant level (p<1×10^-5^). We ensured the independence of these SNPs by applying strict criteria (R^2^ ≤ 0.001; clumping window, 10,000 kb).

### Causal effects between gut microbiota and basal cell carcinoma

76 (genome-wide statistical significance threshold, p < 1×10^−5^) SNPs were selected as IVs and subdivided into two taxa levels: family and genus. Among them, the family classification corresponds to family family XI, which corresponds to 8 SNPs. In the Genus classification, 7 genuses were selected, including Genus Clostridium innocuous group, Genus Family XIII AD3011 group, Genus Paraacteroids, Genus Romboutsia, Genus Ruminiclostridium 5, Genus Ruminococcaceae UCG014, and Genus Tubriciber, which correspond to 7, 12, 6, 13, 10, 10, and 10 SNPs, respectively (**Supplementary Table 1**).

**Figure 3:**
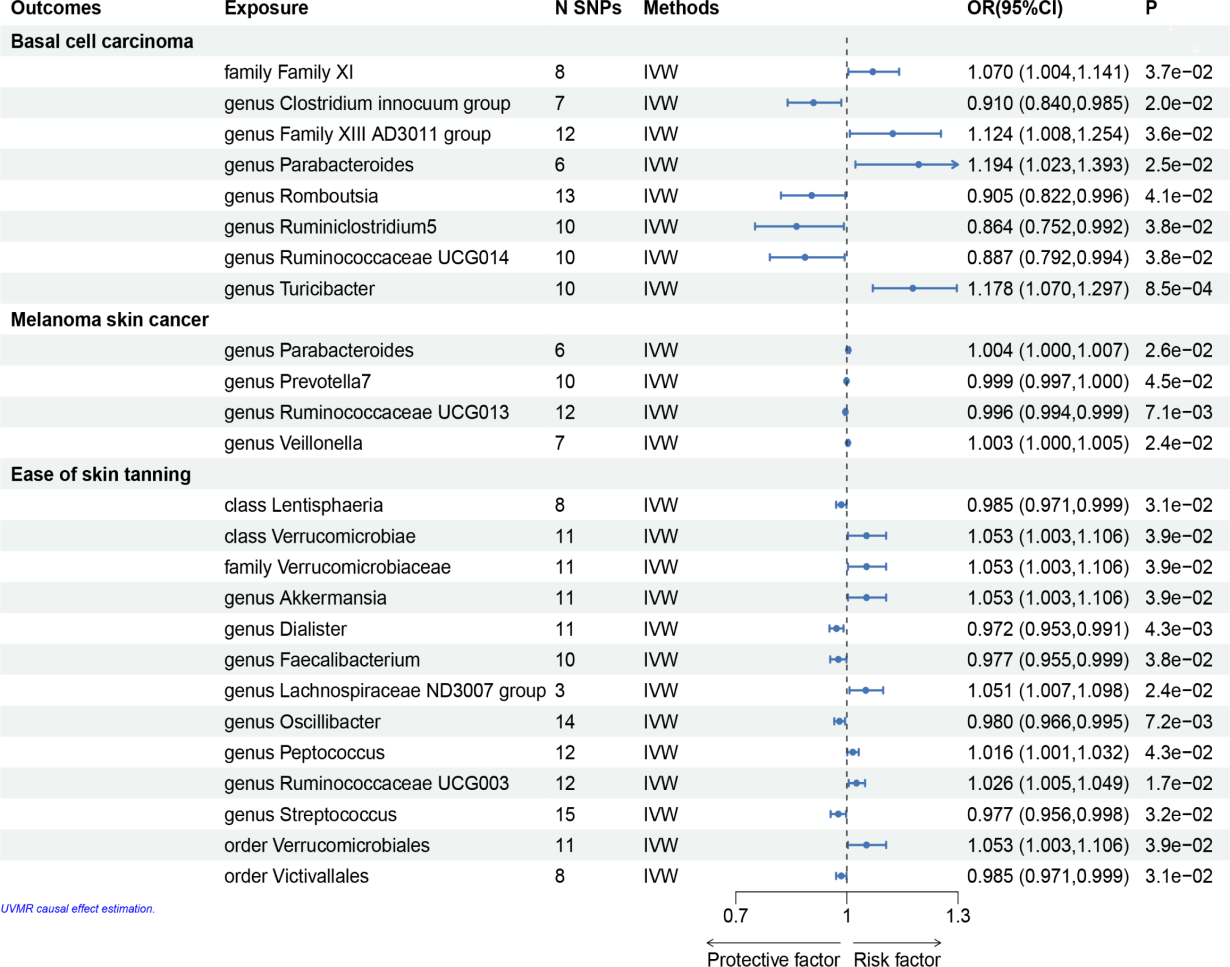
Mendelian randomisation results of causal effects between gut microbiome and basal cell carcinoma, melanoma skin cancer, ease of skin tanning (p<1×10^-5^).

**Figure 4:**
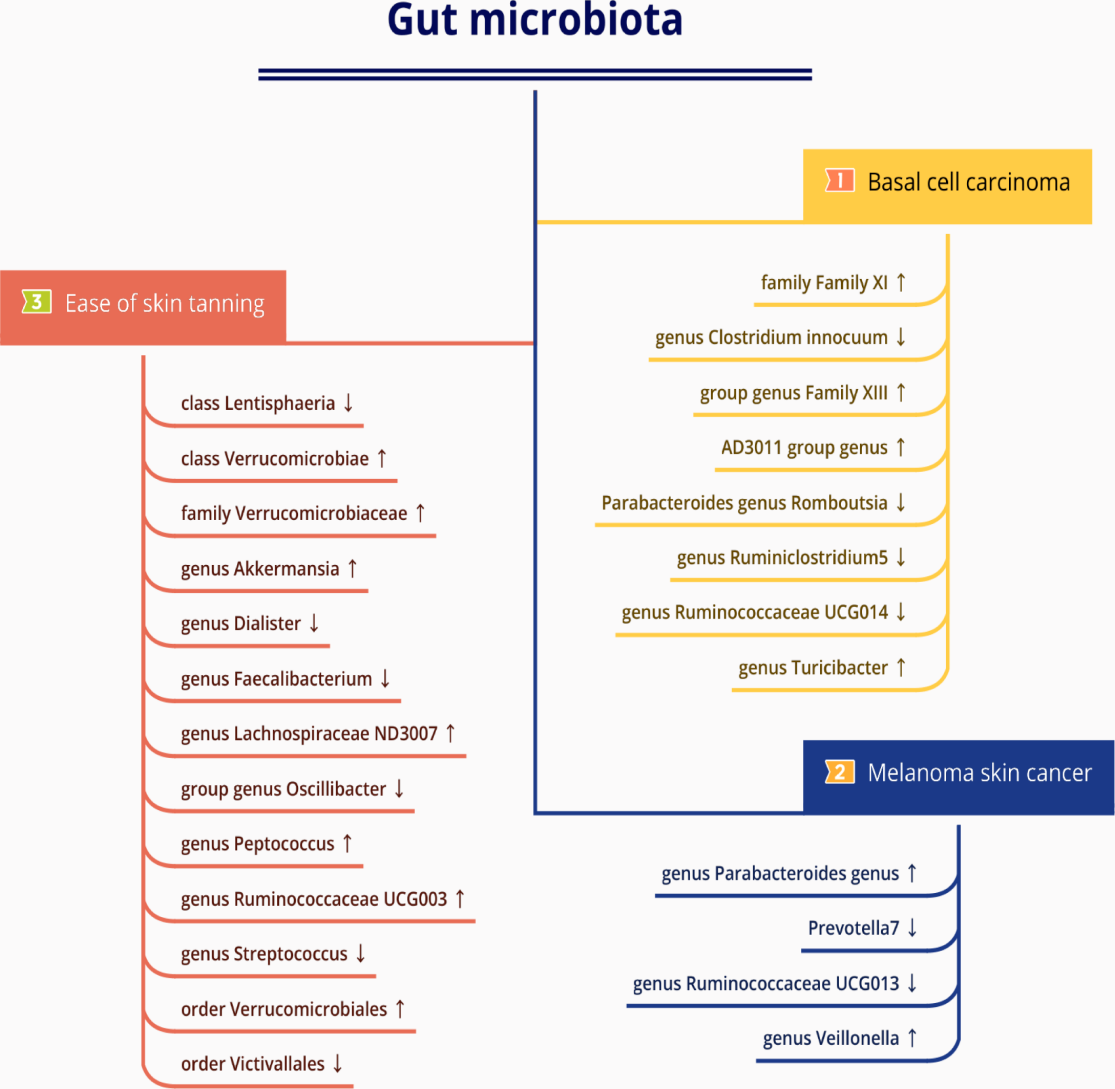
The causal relationships between gut microbiota and basal cell carcinoma, melanoma skin cancer, ease of skin tanning by Mendelian randomisation analysis. Arrow up indicates a positive causal direction between the corresponding microbiota and disease, while arrow down indicates a negative causal direction between the corresponding microbiota and disease.

The F statistics of the IVs were all > 10, indicating no evidence of weak instrument bias. Eventually, after removing pleiotropic SNPs identified by the MR-Egger regression, there was no evidence of horizontal pleiotropy of the IVs (MR Egger regression p > 0.05) (**Supplementary Table 1**, **Figures 3**, **4**).

In the set of IVs, we found that the family Family XI (OR = 1.070, 95% CI = 1.004–1.141, p= 3.7 × 10^−2^, IVW), genus Family XIII AD3011 group (OR = 1.124, 95% CI = 1.008–1.254, p= 3.6 × 10^−2^, IVW), genus Parabacteroides (OR = 1.194, 95% CI = 1.023–1.393, p= 2.5 × 10^−2^, IVW) and genus Turicibacter (OR = 1.178, 95% CI = 1.070–1.297, p= 8.5 × 10^−4^, IVW) causally associated with basal cell carcinoma, it suggests that they may promote the occurrence of this type of carcinoma (**Figures 3**, **4**, **Supplementary Table 1**).

**Figure 5:**
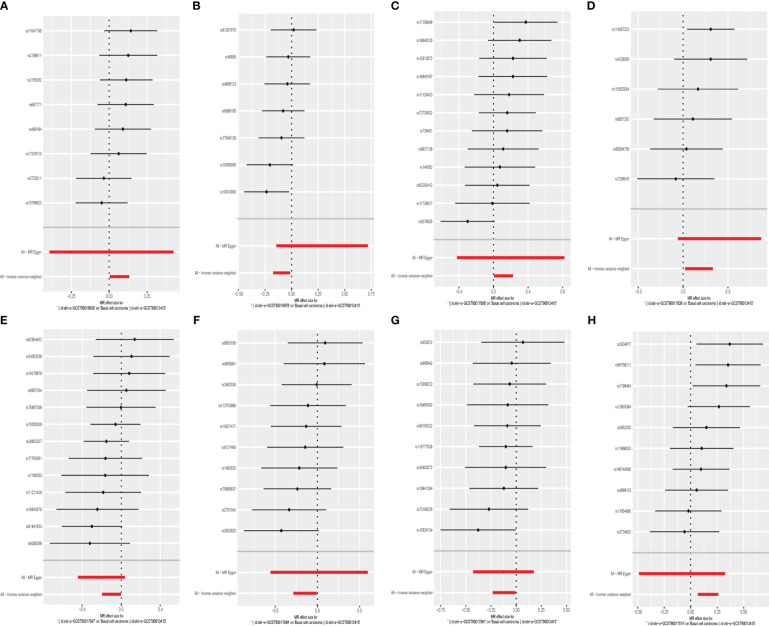
The forest plot represents mendelian randomization results of causal effects between gut microbiome and basal cell carcinoma (p<1×10^-5^). **(A)** Forest plots for the exposure of family Family XI; **(B)** Forest plots for the exposure of genus Clostridium innocuum group; **(C)** Forest plots for the exposure of genus Family XIII AD3011 group; **(D)** Forest plots for the exposure of genus Parabacteroides; **(E)** Forest plots for the exposure of genus Romboutsia; **(F)** Forest plots for the exposure of genus Ruminiclostridium5; **(G)** Forest plots for the exposure of genus Ruminococcaceae UCG014; **(H)** Forest plots for the exposure of genus Turicibacter.

The genus Clostridium innocuum group (OR = 0.910, 95% CI = 0.840–0.985, p = 2.0 × 10^−2^, IVW), Romboutsia (OR = 0.905, 95% CI= 0.822–0.996, p = 4.1 × 10^−2^, IVW), genus Ruminiclostridium5 (OR = 0.864, 95% CI= 0.752–0.992, p = 3.8 × 10^−2^, IVW) and genus Ruminococcaceae UCG014 (OR = 0.887, 95% CI= 0.792–0.994, p = 3.8 × 10^−2^, IVW) were also causally associated with basal cell carcinoma, however, the OR values were all less than 1, which suggested a potential tumor suppressor effect (**Figures 3**–**5**, **Supplementary Table 1**).

### Causal effects between gut microbiota and melanoma skin cancer

MR results for the trait on melanoma skin cancer are shown in **Supplementary Table 2** and **Figure 3**. There are six SNPs in the genus Paraacteroides, 10 SNPs in Genus Prevotella7, 12 SNPs in Genus Ruminococcaceae UCG013 and 7 SNPs in Genus Veillonella.

**Figure 6:**
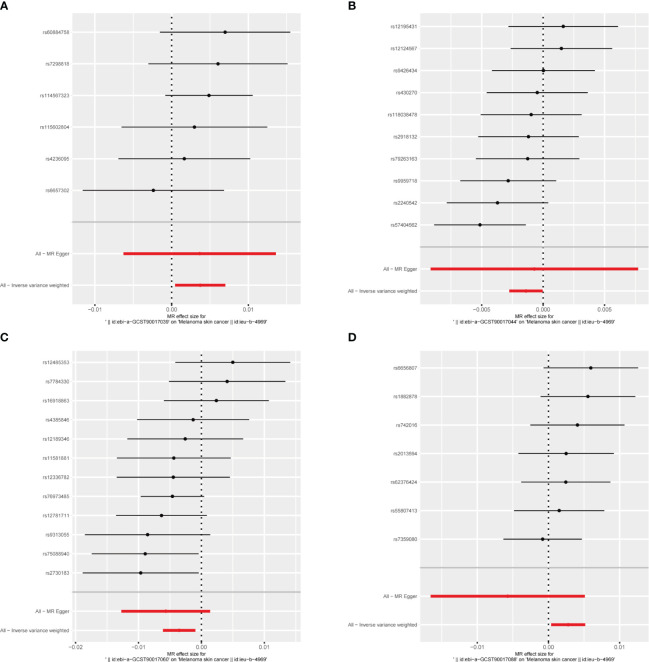
The forest plot represents mendelian randomization results of causal effects between gut microbiome and melanoma skin cancer (p<1×10^-5^). **(A)** Forest plots for the exposure of genus Parabacteroides; **(B)** Forest plots for the exposure of genus Prevotella7; **(C)** Forest plots for the exposure of genus Ruminococcaceae UCG013; **(D)** Forest plots for the exposure of genus Veillonella.

Briefly, among the four genus evaluated in the set of IVs from UK Biobank (p < 1×10^-5^), we found that genetic liability to some gut microbiota was causally associated with melanoma skin cancer, as per the IVW method. we found that the genus Parabacteroides (OR = 1.004, 95% CI = 1.000–1.007, p = 2.6×10^−2^, IVW) and genus Veillonella (OR = 1.003, 95% CI =1.000–1.005, p = 2.4×10^−2^, IVW) and genus Prevotella7 (OR = 1.004, 95% CI = 1.000–1.007, p = 2.6 × 10^−2^, IVW) were causally associated with melanoma skin cancer, but genus Ruminococcaceae UCG013 (OR = 0.996, 95% CI = 0.994–0.999, p = 7.1 × 10^−3^, IVW) showed a negetive causal relationship with melanoma skin cancer (**Figures 3**, **4**, **6**, **Supplementary Table 2**).

### Causal effects between gut microbiota and ease of skin tanning

**Figure 7:**
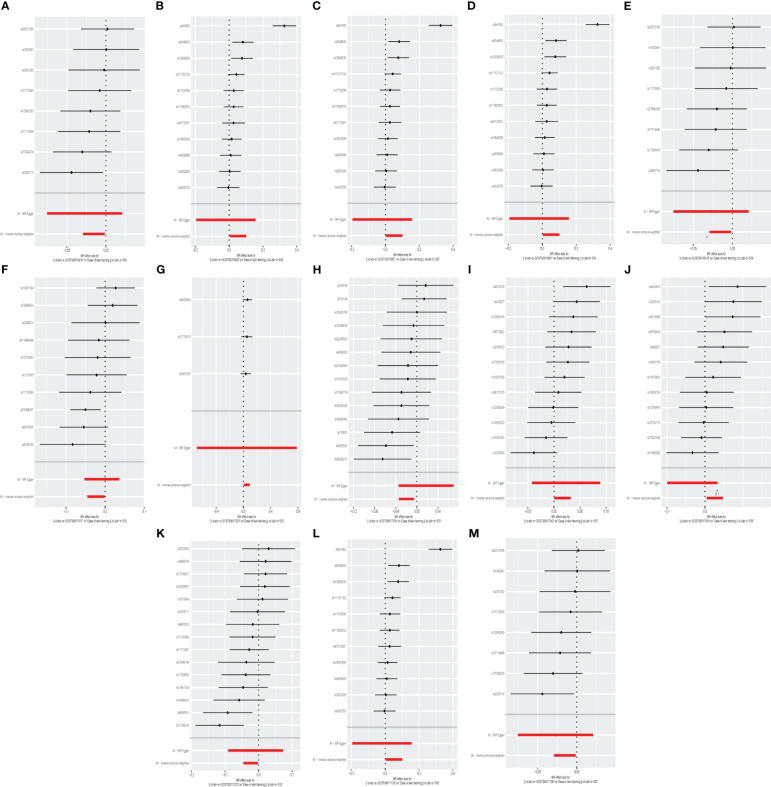
The forest plot represents mendelian randomization results of causal effects between gut microbiome and ease of skin tanning (p<1×10^-5^). **(A)** Forest plots for the exposure of class Lentisphaeria; **(B)** Forest plots for the exposure of class Verrucomicrobiae; **(C)** Forest plots for the exposure of family Verrucomicrobiaceae; **(D)** Forest plots for the exposure of genus Akkermansia; **(E)** Forest plots for the exposure of genus Dialister; **(F)** Forest plots for the exposure of genus Faecalibacterium; **(G)** Forest plots for the exposure of genus Lachnospiraceae ND3007 group; **(H)** Forest plots for the exposure of genus Oscillibacter; **(I)** Forest plots for the exposure of genus Peptococcus; **(J)** Forest plots for the exposure of genus Ruminococcaceae UCG003; **(K)** Forest plots for the exposure of genus Streptococcus; **(L)** Forest plots for the exposure of order Verrucomicrobiales; **(M)** Forest plots for the exposure of order Victivallales.

We identifed 13 gut microbiota related to ease of skin tanning in the other set of IVs (p < 1×10^−5^), which included and subdivide to two classes, one family, eight genuses and two orders from 137 SNPs, we found that the class Verrucomicrobiae (OR = 1.053, 95% CI = 1.003–1.106, p = 3.9×10^−2^, IVW), family Verrucomicrobiaceae (OR = 1.053, 95% CI = 1.003–1.106, p = 3.9×10^−2^, IVW), genus Akkermansia(OR = 1.053, 95% CI = 1.003–1.106, p = 3.9×10^−2^, IVW), genus Lachnospiraceae ND3007 group(OR = 1.051, 95% CI = 1.007–1.098, p = 2.4×10^−2^, IVW), genus Peptococcus(OR = 1.016, 95% CI = 1.001–1.032, p = 4.3×10^−2^, IVW), genus Ruminococcaceae UCG003(OR = 1.026, 95% CI = 1.005–1.049, p = 1.7×10^−2^, IVW) and order Verrucomicrobiales(OR = 1.053, 95% CI = 1.003–1.106, p = 3.9×10^−2^, IVW) were causally positive associated with ease of skin tanning, and we also found that the class Lentisphaeria(OR = 0.985, 95% CI = 0.971–0.999, p = 3.1×10^−2^, IVW), genus Dialister(OR = 0.972, 95% CI = 0.953–0.991, p = 4.3×10^−3^, IVW), genus Faecalibacterium(OR = 0.977, 95% CI = 0.955–0.999, p = 3.8×10^−2^, IVW), genus Oscillibacter(OR = 0.980, 95% CI = 0.966–0.995, p = 7.2×10^−3^, IVW), genus Streptococcus(OR = 0.977, 95% CI = 0.956–0.998, p = 3.2×10^−2^, IVW) and order Victivallales(OR = 0.985, 95% CI = 0.971–0.999, p = 3.1×10^−2^, IVW) were causally negative associated with ease of skin tanning (**Figures 3**, **4**, **7**, **Supplementary Table 3**).

A summary network for a better understanding of the relationship between gut microbiota and cancer is presented in **Figure 4**.

### Sensitivity analyses

The outcomes generated by MR-Egger, Maximum Likelihood, Weighted mode, Simple Mode, and Weighted Median methods provided congruent estimates concerning the intensity and direction of causality. No substantial evidence of horizontal pleiotropy pertaining to gut microbiota in BBC, melanoma skin cancer, and skin tanning susceptibility was manifested with p > 0.05 using the MR-Egger regression intercept approach. Moreover, the findings derived from Cochrane’s Q Statistics denoted lack of significant heterogeneity (p > 0.05) (**Supplementary Table 4**, **Supplementary Materials**).

## Discussion

In this study, using the summary statistics of gut microbiota from the GWAS meta-analysis conducted by the Medical Research Center-Integrative Epidemiology Unit and UK Biobank GWAS Pipeline, we performed three two-sample MR analyses to evaluate the causal association between gut microbiota and basal cell carcinoma, melanoma skin cancer, ease of skin tanning. A total of 25 gut microbiota, including 148 SNPs, were found to be causally associated with basal cell carcinoma, melanoma skin cancer and ease of skin tanning.

Microorganisms inhabiting the gut and other ecological niches potentially contribute to carcinogenesis, mold cancer immune surveillance (41), and dictate responses to immunotherapy, rendering them useful in treating cancer metastasis (42). Through both innate and adaptive immunity, the gut microbiota exerts influence over antitumor immune responses, regulates local and systemic inflammation (43, 44), and enhances the effectiveness of anti-tumor treatments (45). For instance, functions of the gut barrier—including the role of gut microbiota, its integrity, mucus, immune cells, IgA, and antimicrobial peptides (AMPs) created by epithelial cells prevent the intrusion of gut bacteria into the bloodstream, contributing to skin homeostasis maintenance (46, 47). GABA, acetylcholine, dopamine, and serotonin are neurotransmitters generated by the gut microbiota capable of controlling skin function via the nervous system. They can also gain systemic access through gut epithelial and produce wide-ranging effects. Interactions between skin immune cells and microbial communities are not isolated to the local microenvironment. Instead, the skin immune system experiences stimulations from microbial metabolites from elsewhere in the body, including the gut (48). Existing evidence strongly suggests the instrumental role of the gut microbiota in skin cancer development.

### Relationship between the gut microbiome and basal cell carcinoma

Squamous cell carcinoma and basal cell carcinoma, both types of skin cancer, occur in the skin. The link between squamous cell carcinoma and intestinal microbiota has been partially unveiled by current research, but it has only focused on esophageal squamous cell carcinoma and oral squamous cell carcinoma. Family_XI and Ruminococcaceae exist as elements of the Firmicutes phylum. Family_XI is a part of the Clostridiaceae order, with the Family XIII AD3011 group belonging to Family_XI, and Ruminococcaceae UCG014 under Ruminococcaceae. Current understanding states that the normal human gut microbiota comprises two major phyla, including Firmicutes. Research by Duan. et al. (49) have noticed a decrease in the prevalence of Firmicutes in patients suffering from inflammatory bowel disease. Another study (50) identified genetic differences in Clostridium innocuum strains isolated from the intestinal mucosa and mesenteric adipose tissue in patients with Crohn’s disease, suggesting both have significant impacts on intestinal inflammation and immunity. Firmicutes is understood to contribute to the degradation of polysaccharides and the synthesis of essential amino acids (51). Further ex vivo validation of expression patterns (52) suggested that C. innocuum instigates tissue remodeling via M2 macrophages, leading to the formation of an adipose tissue barrier that prevents the systemic dissemination of bacteria. This study discovered positive associations between Family XI, the Family XIII AD3011 group, and basal cell carcinoma. However, the question of whether these groups can promote the growth of carcinoma cells by influencing the migration and transformation of other cells requires further, targeted research for validation.

Doxorubicin, an antitumor antibiotic, can inhibit the synthesis of RNA and DNA, boasting a broad antitumor spectrum effective against various types of tumors. Current research (53) indicates that parabacteroides exhibit protective effects on inflammation and obesity in mice, hinting at their potential therapeutic application in maintaining host-intestine homeostasis. One study (54) discovered that an increase in the abundance of Parabacteroides merdae in the gut and the enhancement of branched-chain amino acid (BCAA) catabolism, triggered by a Ganoderma meroterpene derivative, can combat obesity-associated atherosclerosis. However, there is currently no evidence linking these findings to tissues affected by basal cell carcinoma.

Research analyzing the composition of gut microbiota reveals a notable distinction between psoriasis patients and healthy individuals, with Romboutsia displaying higher relative abundance in the former, suggesting its possible impact on the human immune system response and subsequently the severity of psoriasis (55). Strikingly, no study has explored the connection between Romboutsia and basal cell carcinoma to the best of our knowledge.

Ruminiclostridium5 and Ruminococcaceae UCG014 genera correlate positively with the amounts of butyric and valeric acid in the intestines (56), playing a critical role in maintaining intestinal homeostasis (57, 58). Various experimental results (59–61) suggest that when tumor cell lines are exposed to butyric acid, it can induce cancer cell apoptosis, inhibit cellular proliferation, and promote further differentiation of phenotypes. These multifaceted pathways deliver an anti-angiogenic effect. Additionally, Short-chain fatty acids (SCFAs) pentanoate, and butyrate enhance the anti-tumor activity of cytotoxic T lymphocytes (CTLs) and chimeric antigen receptor (CAR) T cells through metabolic and epigenetic reprogramming (62). This study identifies a negative correlation between these factors and basal cell carcinoma, but further investigation is required to determine whether these SCFA-producing bacterial groups can utilize pentanoate and butyrate to optimize cytotoxic T cells, thereby inhibiting the progression of basal cell carcinoma.

A study (63) unveiled a novel interaction between Turicibacter and bile acids, suggesting that Turicibacter’s strains can elevate the degradation of serum cholesterol, triglycerides, and adipose tissue in mice by influencing the expression of bile modification genes (64). Our research results point towards a positive correlation between Turicibacter and basal cell carcinoma, which begs the question of whether this relationship can be mediated by lipid substances, requiring further investigation.

### Relationship between the gut microbiome and melanoma skin cancer

Recent studies have begun to identify the gut microbiome as a potential new participant in the pathogenesis and treatment of malignant melanoma. In their research, Vitali et al. (65) found that the composition of the gut microbiota in early-stage melanoma transitions from *in situ* to invasive, and finally, to metastatic disease. They observed an abundance of yeasts from the Saccharomytecales order and Prevotella copri species, prevalent in the microbiota of melanoma patients. Concurrently, another study (66) in mice discovered that Lactobacillus reuteri FLRE5K1 could stimulate the production of anti-tumor cell factors, inhibiting the migration of melanoma cells and thereby delaying melanoma onset and extending the subjects’ lifespans. These two studies exemplify the oncogenic and tumor-suppressive roles gut microbiota can play within the body.

The field of cancer immunotherapy has witnessed significant breakthroughs in recent years, such as the developments in understanding cancer immune checkpoints and the progression of Immune Checkpoint Inhibitors (ICIs). These advances have revolutionized melanoma treatment. However, recent research has brought the gut microbiota into this dynamic equation. Numerous projects are currently exploring the potential of altering the intestinal microbiome’s composition, particularly through fecal microbial transplantation (FMT). It has been widely proven that this method can overcome resistance to checkpoint inhibitor therapy in malignant melanoma, and reintroduce a clinical reaction post-FMT (67, 68).

An *in vivo* study’s results (45) show that the gut microbiome could possibly alter responses to anti PD-1 immunotherapy in melanoma patients. Yet, the results of our study differ somewhat from these findings: we discerned a likely positive connection between the Prevotella7 genus and the incidence of melanoma; our results concerning Ruminococcaceae, on the other hand, align with theirs, as the Ruminococcaceae UCG013 genus demonstrated a negative causal link with melanoma skin cancer, suggesting it has an anti-tumor potential. Previous study (69) reported that Ruminococcus bromii potentially enhances antitumor responses to Immune Checkpoint Inhibitor (ICI). Gopalakrishnan and colleagues further found a correlation between heightened faecal levels of the Clostridiales family, specifically Ruminococcaceae, and improved response rates in patients undergoing anti-PD-1 therapy. They applied techniques like 16S rRNA gene sequencing for these observations. It was also revealed that, when complemented with Faecal Microbiota Transplantation (FMT), germ-free mice showed a notable increase in intra-tumoral CD8+ T cell count and decreased melanoma growth in response to anti-PD-1 therapy (21). In a recent meta-analysis that included 130 patients from four studies, Limeta et al. (70) found an overrepresentation of the Faecalibacterium taxa to be beneficial, similar to the presence of Ruminococcacea and Barnesiella intestinihominis. These observations emphasize the potential therapeutic advantages of modulating the gut microbiome in patients receiving checkpoint blockade immunotherapy, necessitating immediate evaluation in cancer patients via clinical trials.

In a different study, Wu et al. (71) gathered stool samples from cancer patients undergoing anti-PD-1 and chemotherapy combination treatment for fecal metagenomic sequencing. Through comparing microbiota diversity and composition amongst the responder and non-responder groups. They determined that the Parabacteroides genus was more prolific in the responder group at the initial stage. Based on our findings, Parabacteroides is positively associated with melanoma promotion, and thus, might serve as a potential research point impacting the anti-PD-1 treatment for melanoma.

Moreover, Lee et al. (72) detected a significant difference in fecal bacteria between patients with radiology-confirmed objective responses and patients with progressive disease prior to immunotherapy. They observed a predominance of Veillonella in patients with radiology-verified objective responses, significantly contrasting our results. They also noticed an enrichment of Prevotella 9 in patients with progressive disease—for which depletion predicted better overall survival in subsequent experiments. These findings correspond with our research results that suggest an enriched Prevotella7 genus might play a potential role in melanoma development.

Despite the advances, specific interaction investigations are still in exploratory stages, but they have started accruing momentum following the emergence of more substantial and clinically pertinent effects (73). In-depth functional analyses on both community and per-microbe scales will likely be necessary to clarify microbial-immune-cancer cell mechanistic interactions. Simultaneously, effective methods of isolating components that deal with beneficial bacteria should be sought to enable absorption to replace fecal transplantation of certain bacterial membrane proteins (74).

### Relationship between the gut microbiome and ease of skin tanning

Exposure to ultraviolet (UV) rays can incite inflammation (75) and has been found to modify both local (skin) and systemic (intestinal) microbiomes (76). This UV-induced damage to the immune system hampers the host’s capacity to counteract skin cancer, thereby promoting carcinogenesis (77). Cumulative exposure to UV rays is commonly linked with BCC and squamous cell carcinoma (SCC) (78), which are prevalent types of cancer in European populations, particularly among individuals with fair skin (27). In the realm of cancer immunobiology, microbes play a critical role by curbing the evolution of chronic inflammation during the initial stages (79). The body’s response to tanning after sun exposure, primarily governed by melanin pigmentation, is protective against DNA photodamage. However, the tanning response exhibits significant variability, both within and across populations. An estimate from the UK Biobank sample (80) suggests that, due to prevalent genetic variations, the heritability of the ease of skin tanning is around 0.454 ± 0.006. Disturbances in the homeostasis of skin microbiomes may instigate inflammatory mechanisms potentially leading to cancer. Recent studies have shown a higher relative abundance of symbiotic bacterial strains in non-lesional skin compared to skin affected by actinic keratosis (AK) and SCC. For example, Staphylococcus aureus is markedly higher in AK and SCC lesions (81, 82).

Oral probiotics have shown potential for controlling UV-B-induced immunosuppression, and lipospheric acid of Lactobacillus reuteri, when administered orally, has found to reduce the quantity of UV-induced skin tumors in SKH-1 hairless mice (83). Oral prebiotics can either stimulate or restrain the proliferation of specific gut microbes, and it is now thought to regulate the growth of certain harmful skin microbes as well. Feeding mice with mixed probiotics has resulted in gut microbiome modulation and mitigation of UVB-caused skin aging by downregulating the MAPK pathway (84). Moreover, some studies (85, 86) have reported that oral probiotics can alleviate skin inflammation in mice with skin conditions. In this study, we have discovered that the Lentisphaeria class, Dialister genus, Faecalibacterium genus, Oscillibacter genus, Streptococcus genus, and Victivallales order, all related to the ease of skin tanning, were found to have a causal negative correlation with ease of skin tanning. Conversely, the Verrucomicrobiae class, Verrucomicrobiaceae family, Akkermansia genus, Lachnospiraceae ND3007 group, Peptococcus genus, Ruminococcaceae UCG003 genus, and Verrucomicrobiales order displayed a causal positive correlation with ease of skin tanning. These findings suggest that tanning, microbial groups, and skin cancer may all be interconnected. Therefore, future research should focus on elucidating these intrinsic relationships more clearly.

### Advantages and disadvantages

This study has several strengths. MR analysis was performed to determine the causal association between gut microbiota and basal cell carcinoma, melanoma skin cancer, ease of skin tanning, thus excluding the interference of confounding factors. Genetic variants of gut microbiota were obtained from the largest available GWAS meta-analysis, ensuring the strength of instruments in the MR analysis. Horizontal pleiotropy was detected and excluded by using the IVW and MR-Egger regression intercept term tests. Furthermore, Maximum Likelihood, Weighted mode, Simple Mode, Weighted Median and Cochrane’s Q Statistics were used to rule out the bias and heterogeneity. A two-sample MR design was adopted and non-overlapping exposure and outcome summary-level data were used to avoid bias.

However, there are also several limitations in this study, which should be noted while interpreting the results. Because summary statistics rather than raw data were used in the analysis, it was not possible to perform subgroup analyses, such as distinguishing different types of basal cell carcinoma and melanoma skin cancer, or exploring non-linear relationships. To conduct sensitivity analysis and horizontal pleiotropy detection, more genetic variations need to be included as instrumental variables; therefore, SNP used in the analysis did not reach the traditional GWAS significance threshold (P < 5×10^–8^), which may increase the possibility of false positives. The sample size of gut microbiota was relatively small, so reverse MR analysis was not applied to further explore the reverse causal association, which could not be completely excluded. Although most participants in the GWAS meta-analysis for gut microbiota data were of European descent, there may still be interference from population stratification, and the results of this study may not be entirely applicable to subjects of non-European descent. Future MR studies on the causal association between gut microbiota and basal cell carcinoma, melanoma skin cancer, ease of skin tanning could be considered in diverse European and non-European populations for better generalizability. Furthermore, the Clustered Regular Interspace Short Palindromic Repeats (CRISPR) - based “active genetic” elements developed in 2015 (87) passed the fundamental rules of traditional genetics, as easily accessible and programmable tools for gene editing and regulation can be actively selected to identify the genes our mendelian randomization study was unable to utilize this tool to validate causal relationships and make this prediction to take to the next level at the molecular level. Future research will focus on resolving this defect.

## Conclusions

In summary, the results of three two-sample Mendelian randomization studies found that the gut microbiome was causally associated with basal cell carcinoma, melanoma skin cancer and ease of skin tanning. Further randomized controlled trials are needed to clarify the effect of the gut microbiome on basal cell carcinoma, melanoma skin cancer, ease of skin tanning and their specific mechanisms. In addition, reverse MR was not used in this study to support the causal association between the gut microbiome and basal cell carcinoma, melanoma skin cancer, ease of skin tanning, this again needs to be confirmed by further studies.

## Data availability statement

The original contributions presented in the study are included in the article/**Supplementary Materials**, further inquiries can be directed to the corresponding author.

## Ethics statement

Ethical approval was not required for the study involving humans in accordance with the local legislation and institutional requirements. Written informed consent to participate in this study was not required from the participants or the participants’ legal guardians/next of kin in accordance with the national legislation and the institutional requirements.

## Author contributions

JQL: Methodology, Conceptualization, Investigation, Data curation, Formal Analysis, Software, Writing – original draft, Writing – review & editing. SC: Conceptualization, Investigation, Writing – review & editing, Validation. JLL: Formal Analysis, Methodology, Software, Writing – review & editing, Supervision. GJ: Formal Analysis, Supervision, Investigation, Writing – review & editing. YF: Formal Analysis, Funding acquisition, Project administration, Resources, Validation, Visualization, Writing – review & editing. NH: Resources, Supervision, Methodology, Writing – original draft, Writing – review & editing.
