# Supplementary material for: Causal relationship between the gut microbiome and basal cell carcinoma, melanoma skin cancer, ease of skin tanning: evidence from three two-sample mendelian randomisation studies
Source: Front Immunol. 2024 Jan 18;15:1279680. doi: 10.3389/fimmu.2024.1279680 (PMC10830803; doi:10.3389/fimmu.2024.1279680)
Supplement: Supplementary file 13 [file Table_1.docx]

**Supplementary Table 1. Information on IVs exposed when gut microbiota is exposed and basal cell carcinoma is the outcome.**

| Exposure | SNP | A1 | A2 | BETA | SE | EAF | P | R^2^ | F_statistics |
| --- | --- | --- | --- | --- | --- | --- | --- | --- | --- |
| family Family XI | rs10759623 | C | T | -0.162 | 0.032 | 0.177 | 5.78E-07 | 0.001774 | 25.42 |
| family Family XI | rs11547158 | A | G | -0.178 | 0.037 | 0.138 | 2.70E-06 | 0.001584 | 22.69 |
| family Family XI | rs17379710 | T | C | -0.116 | 0.025 | 0.443 | 3.97E-06 | 0.001487 | 21.31 |
| family Family XI | rs2155352 | A | G | -0.151 | 0.030 | 0.215 | 6.63E-07 | 0.00173 | 24.79 |
| family Family XI | rs2156611 | T | C | -0.112 | 0.025 | 0.479 | 9.43E-06 | 0.001401 | 20.07 |
| family Family XI | rs3733511 | A | G | 0.128 | 0.027 | 0.299 | 3.39E-06 | 0.001521 | 21.79 |
| family Family XI | rs488164 | G | T | -0.118 | 0.026 | 0.605 | 4.80E-06 | 0.001493 | 21.38 |
| family Family XI | rs697771 | A | G | -0.118 | 0.025 | 0.435 | 3.19E-06 | 0.001528 | 21.89 |
| genus Clostridium innocuum group | rs10074000 | T | C | -0.103 | 0.023 | 0.403 | 7.00E-06 | 0.001421 | 20.35 |
| genus Clostridium innocuum group | rs10506058 | A | G | 0.1 | 0.022 | 0.406 | 8.92E-06 | 0.001409 | 20.18 |
| genus Clostridium innocuum group | rs40656 | C | T | 0.143 | 0.031 | 0.161 | 8.62E-06 | 0.001469 | 21.04 |
| genus Clostridium innocuum group | rs4869133 | G | A | -0.181 | 0.041 | 0.081 | 7.24E-06 | 0.001358 | 19.45 |
| genus Clostridium innocuum group | rs61267978 | T | C | 0.147 | 0.032 | 0.131 | 5.59E-06 | 0.001466 | 21.01 |
| genus Clostridium innocuum group | rs6890185 | C | T | -0.113 | 0.023 | 0.671 | 1.12E-06 | 0.001652 | 23.67 |
| genus Clostridium innocuum group | rs77845139 | A | G | -0.115 | 0.026 | 0.257 | 8.41E-06 | 0.001395 | 19.99 |
| genus Family XIII AD3011 group | rs11126423 | T | C | -0.09 | 0.020 | 0.904 | 5.91E-06 | 0.001482 | 21.22 |
| genus Family XIII AD3011 group | rs11736617 | G | A | -0.076 | 0.017 | 0.120 | 9.02E-06 | 0.001359 | 19.47 |
| genus Family XIII AD3011 group | rs12812672 | T | C | -0.096 | 0.021 | 0.090 | 2.56E-06 | 0.001485 | 21.27 |
| genus Family XIII AD3011 group | rs149302 | T | C | -0.061 | 0.014 | 0.209 | 7.48E-06 | 0.001418 | 20.32 |
| genus Family XIII AD3011 group | rs16840310 | G | A | 0.061 | 0.012 | 0.643 | 6.75E-07 | 0.001729 | 24.78 |
| genus Family XIII AD3011 group | rs16940167 | C | T | 0.073 | 0.016 | 0.156 | 3.91E-06 | 0.001465 | 20.99 |
| genus Family XIII AD3011 group | rs17156849 | G | A | -0.113 | 0.025 | 0.074 | 4.19E-06 | 0.001478 | 21.18 |
| genus Family XIII AD3011 group | rs62200412 | C | T | -0.08 | 0.016 | 0.158 | 5.80E-07 | 0.001667 | 23.89 |
| genus Family XIII AD3011 group | rs72730932 | C | A | -0.09 | 0.018 | 0.156 | 6.89E-07 | 0.0018 | 25.79 |
| genus Family XIII AD3011 group | rs739451 | C | T | 0.065 | 0.015 | 0.201 | 7.88E-06 | 0.001353 | 19.38 |
| genus Family XIII AD3011 group | rs9276029 | A | G | -0.081 | 0.019 | 0.220 | 8.93E-06 | 0.001333 | 19.09 |
| genus Family XIII AD3011 group | rs9837139 | A | G | 0.108 | 0.024 | 0.063 | 8.71E-06 | 0.001395 | 19.99 |
| genus Parabacteroides | rs114567323 | T | C | 0.186 | 0.041 | 0.046 | 5.65E-06 | 0.001478 | 21.18 |
| genus Parabacteroides | rs115602804 | G | A | 0.103 | 0.022 | 0.055 | 1.93E-06 | 0.001495 | 21.41 |
| genus Parabacteroides | rs4236095 | A | G | -0.076 | 0.016 | 0.864 | 1.93E-06 | 0.001643 | 23.54 |
| genus Parabacteroides | rs60884758 | C | T | -0.07 | 0.014 | 0.171 | 5.71E-07 | 0.001703 | 24.4 |
| genus Parabacteroides | rs6657302 | T | C | -0.105 | 0.023 | 0.058 | 9.76E-06 | 0.001499 | 21.48 |
| genus Parabacteroides | rs7298818 | C | T | 0.089 | 0.020 | 0.085 | 8.54E-06 | 0.001366 | 19.57 |
| genus Romboutsia | rs10279978 | A | G | -0.062 | 0.013 | 0.312 | 1.17E-06 | 0.001657 | 23.75 |
| genus Romboutsia | rs11221428 | T | C | -0.073 | 0.016 | 0.171 | 6.49E-06 | 0.001472 | 21.09 |
| genus Romboutsia | rs16843578 | C | T | -0.088 | 0.020 | 0.090 | 5.08E-06 | 0.001381 | 19.78 |
| genus Romboutsia | rs28603357 | T | C | -0.215 | 0.047 | 0.046 | 8.52E-06 | 0.00143 | 20.49 |
| genus Romboutsia | rs34302036 | G | A | -0.055 | 0.012 | 0.581 | 5.88E-06 | 0.001447 | 20.73 |
| genus Romboutsia | rs61841503 | G | A | 0.093 | 0.017 | 0.169 | 4.00E-08 | 0.002047 | 29.35 |
| genus Romboutsia | rs62504452 | A | G | -0.071 | 0.016 | 0.173 | 4.66E-06 | 0.001434 | 20.54 |
| genus Romboutsia | rs7109293 | A | G | 0.092 | 0.021 | 0.074 | 6.98E-06 | 0.001393 | 19.95 |
| genus Romboutsia | rs75200530 | T | G | -0.191 | 0.042 | 0.050 | 5.07E-06 | 0.001431 | 20.5 |
| genus Romboutsia | rs75987356 | G | A | -0.13 | 0.028 | 0.059 | 6.71E-06 | 0.00149 | 21.34 |
| genus Romboutsia | rs77702691 | A | G | -0.094 | 0.021 | 0.099 | 7.37E-06 | 0.00143 | 20.49 |
| genus Romboutsia | rs9389266 | T | G | 0.072 | 0.016 | 0.154 | 9.38E-06 | 0.001384 | 19.82 |
| genus Romboutsia | rs9567264 | C | T | 0.058 | 0.013 | 0.302 | 5.76E-06 | 0.001445 | 20.69 |
| genus Ruminiclostridium5 | rs10827477 | A | G | -0.055 | 0.012 | 0.320 | 2.19E-06 | 0.001577 | 22.59 |
| genus Ruminiclostridium5 | rs113753996 | T | C | 0.082 | 0.017 | 0.093 | 3.99E-06 | 0.001544 | 22.13 |
| genus Ruminiclostridium5 | rs1492620 | T | C | -0.083 | 0.018 | 0.090 | 3.53E-06 | 0.001485 | 21.27 |
| genus Ruminiclostridium5 | rs2482038 | C | A | 0.052 | 0.011 | 0.432 | 1.70E-06 | 0.001589 | 22.76 |
| genus Ruminiclostridium5 | rs2791343 | T | C | 0.052 | 0.011 | 0.329 | 5.54E-06 | 0.001453 | 20.81 |
| genus Ruminiclostridium5 | rs2833828 | G | A | 0.05 | 0.011 | 0.428 | 6.82E-06 | 0.001416 | 20.29 |
| genus Ruminiclostridium5 | rs4955951 | A | G | -0.072 | 0.017 | 0.109 | 9.96E-06 | 0.001293 | 18.52 |
| genus Ruminiclostridium5 | rs6121460 | G | A | 0.093 | 0.020 | 0.070 | 2.64E-06 | 0.001531 | 21.93 |
| genus Ruminiclostridium5 | rs79968837 | A | G | -0.095 | 0.019 | 0.084 | 1.15E-06 | 0.001683 | 24.11 |
| genus Ruminiclostridium5 | rs8053158 | G | A | 0.074 | 0.016 | 0.879 | 5.90E-06 | 0.001511 | 21.65 |
| genus Ruminococcaceae UCG014 | rs10495392 | T | C | 0.082 | 0.019 | 0.886 | 9.96E-06 | 0.001355 | 19.41 |
| genus Ruminococcaceae UCG014 | rs10941294 | C | T | -0.122 | 0.026 | 0.069 | 2.40E-06 | 0.001538 | 22.03 |
| genus Ruminococcaceae UCG014 | rs115777838 | T | C | -0.188 | 0.039 | 0.047 | 4.62E-07 | 0.001656 | 23.73 |
| genus Ruminococcaceae UCG014 | rs12638134 | T | G | 0.058 | 0.012 | 0.402 | 1.21E-06 | 0.001654 | 23.7 |
| genus Ruminococcaceae UCG014 | rs34402072 | C | T | -0.069 | 0.016 | 0.185 | 9.80E-06 | 0.001356 | 19.43 |
| genus Ruminococcaceae UCG014 | rs56105232 | G | A | 0.139 | 0.030 | 0.058 | 2.91E-06 | 0.001513 | 21.68 |
| genus Ruminococcaceae UCG014 | rs72809222 | T | C | 0.067 | 0.014 | 0.261 | 2.41E-06 | 0.001611 | 23.07 |
| genus Ruminococcaceae UCG014 | rs73186226 | G | A | -0.099 | 0.022 | 0.081 | 6.72E-06 | 0.001467 | 21.01 |
| genus Ruminococcaceae UCG014 | rs853612 | A | G | -0.053 | 0.012 | 0.403 | 9.75E-06 | 0.001367 | 19.58 |
| genus Ruminococcaceae UCG014 | rs995642 | C | T | 0.06 | 0.013 | 0.302 | 1.90E-06 | 0.001575 | 22.56 |
| genus Turicibacter | rs11054680 | T | C | -0.105 | 0.023 | 0.120 | 2.31E-06 | 0.001486 | 21.29 |
| genus Turicibacter | rs11666533 | C | T | -0.112 | 0.025 | 0.114 | 7.37E-06 | 0.001411 | 20.21 |
| genus Turicibacter | rs12603364 | T | C | 0.111 | 0.023 | 0.118 | 8.67E-07 | 0.001685 | 24.14 |
| genus Turicibacter | rs149744580 | A | G | 0.17 | 0.032 | 0.058 | 7.01E-08 | 0.002023 | 28.99 |
| genus Turicibacter | rs2834977 | T | C | -0.096 | 0.021 | 0.150 | 3.96E-06 | 0.001483 | 21.25 |
| genus Turicibacter | rs2952020 | A | G | 0.076 | 0.017 | 0.725 | 5.63E-06 | 0.001463 | 20.96 |
| genus Turicibacter | rs3734633 | G | A | -0.121 | 0.027 | 0.080 | 5.32E-06 | 0.001419 | 20.32 |
| genus Turicibacter | rs4869133 | G | A | 0.131 | 0.027 | 0.081 | 2.55E-06 | 0.001624 | 23.26 |
| genus Turicibacter | rs55756211 | T | C | -0.115 | 0.024 | 0.104 | 2.81E-06 | 0.001596 | 22.87 |
| genus Turicibacter | rs7199484 | G | A | -0.073 | 0.016 | 0.308 | 5.77E-06 | 0.001456 | 20.85 |

A1: gut microbiome increasing allele; A2: other allele; SE: standard error; EAF: effect allele frequency
