# Supplementary material for: Causal relationship between the gut microbiome and basal cell carcinoma, melanoma skin cancer, ease of skin tanning: evidence from three two-sample mendelian randomisation studies
Source: Front Immunol. 2024 Jan 18;15:1279680. doi: 10.3389/fimmu.2024.1279680 (PMC10830803; doi:10.3389/fimmu.2024.1279680)
Supplement: Supplementary file 14 [file Table_2.docx]

**Supplementary Table 2. Information on IVs exposed when gut microbiota is exposed and melanoma skin cancer is the outcome.**

| Exposure | SNP | A1 | A2 | BETA | SE | EAF | P | R^2^ | F_statistics |
| --- | --- | --- | --- | --- | --- | --- | --- | --- | --- |
| genus Parabacteroides | rs114567323 | T | C | 0.186 | 0.041 | 0.048 | 5.65E-06 | 0.001478284 | 21.18 |
| genus Parabacteroides | rs115602804 | G | A | 0.103 | 0.022 | 0.056 | 1.93E-06 | 0.001494858 | 21.41 |
| genus Parabacteroides | rs4236095 | A | G | -0.076 | 0.016 | 0.860 | 1.93E-06 | 0.001642864 | 23.54 |
| genus Parabacteroides | rs60884758 | C | T | -0.070 | 0.014 | 0.175 | 5.71E-07 | 0.001702716 | 24.40 |
| genus Parabacteroides | rs6657302 | T | C | -0.105 | 0.023 | 0.058 | 9.76E-06 | 0.001499184 | 21.48 |
| genus Parabacteroides | rs7298818 | C | T | 0.089 | 0.020 | 0.085 | 8.54E-06 | 0.00136634 | 19.57 |
| genus Prevotella7 | rs118038478 | A | G | 0.206 | 0.047 | 0.078 | 7.85E-06 | 0.001342992 | 19.24 |
| genus Prevotella7 | rs12124567 | A | G | -0.121 | 0.028 | 0.289 | 9.49E-06 | 0.001357558 | 19.44 |
| genus Prevotella7 | rs12195431 | T | C | 0.197 | 0.044 | 0.075 | 8.73E-06 | 0.001378017 | 19.74 |
| genus Prevotella7 | rs2240542 | C | T | 0.121 | 0.026 | 0.284 | 4.84E-06 | 0.001487541 | 21.31 |
| genus Prevotella7 | rs2918132 | T | C | 0.115 | 0.025 | 0.642 | 6.42E-06 | 0.00141191 | 20.22 |
| genus Prevotella7 | rs430270 | A | C | 0.139 | 0.030 | 0.204 | 2.87E-06 | 0.001531581 | 21.94 |
| genus Prevotella7 | rs57404562 | C | A | 0.155 | 0.032 | 0.188 | 6.22E-07 | 0.001688883 | 24.20 |
| genus Prevotella7 | rs79263163 | A | C | -0.144 | 0.032 | 0.166 | 7.51E-06 | 0.001458195 | 20.89 |
| genus Prevotella7 | rs9426434 | T | C | -0.124 | 0.028 | 0.267 | 9.72E-06 | 0.001376028 | 19.71 |
| genus Prevotella7 | rs9959718 | G | A | 0.133 | 0.028 | 0.255 | 1.90E-06 | 0.001628361 | 23.33 |
| genus Ruminococcaceae UCG013 | rs11581881 | C | T | 0.066 | 0.014 | 0.177 | 4.73E-06 | 0.001456719 | 20.87 |
| genus Ruminococcaceae UCG013 | rs12189346 | G | A | 0.068 | 0.015 | 0.149 | 1.68E-06 | 0.001545036 | 22.13 |
| genus Ruminococcaceae UCG013 | rs12336782 | T | C | -0.086 | 0.019 | 0.100 | 8.60E-06 | 0.001427266 | 20.44 |
| genus Ruminococcaceae UCG013 | rs12485353 | G | A | -0.061 | 0.013 | 0.208 | 4.19E-06 | 0.001506496 | 21.58 |
| genus Ruminococcaceae UCG013 | rs12781711 | C | T | -0.066 | 0.012 | 0.346 | 2.55E-08 | 0.002175748 | 31.19 |
| genus Ruminococcaceae UCG013 | rs16918863 | A | C | 0.111 | 0.024 | 0.062 | 4.15E-06 | 0.00150424 | 21.55 |
| genus Ruminococcaceae UCG013 | rs2730183 | G | A | -0.049 | 0.011 | 0.470 | 8.44E-06 | 0.001380072 | 19.77 |
| genus Ruminococcaceae UCG013 | rs4385846 | T | G | -0.060 | 0.013 | 0.768 | 6.46E-06 | 0.001438694 | 20.61 |
| genus Ruminococcaceae UCG013 | rs75088940 | T | C | -0.094 | 0.020 | 0.091 | 2.55E-06 | 0.001540492 | 22.07 |
| genus Ruminococcaceae UCG013 | rs76973485 | G | T | 0.195 | 0.042 | 0.056 | 3.35E-06 | 0.001517014 | 21.73 |
| genus Ruminococcaceae UCG013 | rs7784330 | A | G | 0.050 | 0.011 | 0.612 | 8.16E-06 | 0.001380278 | 19.77 |
| genus Ruminococcaceae UCG013 | rs9313055 | T | C | 0.105 | 0.023 | 0.049 | 9.55E-06 | 0.001402274 | 20.09 |
| genus Veillonella | rs1882878 | A | G | -0.077 | 0.016 | 0.266 | 2.98E-06 | 0.00153613 | 22.01 |
| genus Veillonella | rs2013594 | C | T | 0.072 | 0.016 | 0.672 | 3.42E-06 | 0.00150596 | 21.57 |
| genus Veillonella | rs55807413 | A | G | 0.107 | 0.024 | 0.126 | 5.51E-06 | 0.001423913 | 20.40 |
| genus Veillonella | rs62376424 | C | T | -0.076 | 0.016 | 0.343 | 3.65E-06 | 0.00151687 | 21.73 |
| genus Veillonella | rs6656807 | G | A | -0.070 | 0.015 | 0.596 | 5.50E-06 | 0.001455642 | 20.85 |
| genus Veillonella | rs7359080 | C | A | -0.135 | 0.030 | 0.125 | 7.40E-06 | 0.001388939 | 19.90 |
| genus Veillonella | rs742016 | A | G | -0.069 | 0.015 | 0.423 | 4.66E-06 | 0.001475347 | 21.13 |

A1: gut microbiome increasing allele; A2: other allele; SE: standard error; EAF: effect allele frequency
