# Supplementary material for: Causal relationship between the gut microbiome and basal cell carcinoma, melanoma skin cancer, ease of skin tanning: evidence from three two-sample mendelian randomisation studies
Source: Front Immunol. 2024 Jan 18;15:1279680. doi: 10.3389/fimmu.2024.1279680 (PMC10830803; doi:10.3389/fimmu.2024.1279680)
Supplement: Supplementary file 15 [file Table_3.docx]

**Supplementary Table 3. Information on IVs exposed when gut microbiota is exposed and ease of skin tanning is the outcome.**

| Exposure | SNP | A1 | A2 | BETA | SE | EAF | P | R^2^ | F_statistics |
| --- | --- | --- | --- | --- | --- | --- | --- | --- | --- |
| class Lentisphaeria | rs1002941 | A | G | -0.105 | 0.023 | 0.726 | 8.15E-06 | 0.001412339 | 20.23 |
| class Lentisphaeria | rs11770843 | C | T | 0.109 | 0.023 | 0.291 | 1.91E-06 | 0.001515011 | 21.70 |
| class Lentisphaeria | rs17114848 | G | A | 0.152 | 0.032 | 0.123 | 4.06E-06 | 0.001540535 | 22.07 |
| class Lentisphaeria | rs2031282 | A | G | 0.122 | 0.027 | 0.171 | 4.38E-06 | 0.001430246 | 20.49 |
| class Lentisphaeria | rs2825714 | A | G | -0.137 | 0.029 | 0.147 | 1.72E-06 | 0.001575067 | 22.57 |
| class Lentisphaeria | rs62570196 | C | T | -0.216 | 0.044 | 0.080 | 1.08E-06 | 0.001688189 | 24.19 |
| class Lentisphaeria | rs72640280 | A | G | 0.220 | 0.049 | 0.058 | 5.18E-06 | 0.001431855 | 20.51 |
| class Lentisphaeria | rs77599476 | A | G | 0.230 | 0.048 | 0.055 | 1.86E-06 | 0.001605297 | 23.00 |
| class Verrucomicrobiae | rs111862613 | T | C | 0.091 | 0.020 | 0.171 | 3.74E-06 | 0.001483304 | 21.25 |
| class Verrucomicrobiae | rs117107102 | A | G | 0.205 | 0.043 | 0.042 | 2.92E-06 | 0.001569844 | 22.49 |
| class Verrucomicrobiae | rs11729256 | T | C | 0.075 | 0.015 | 0.259 | 6.73E-07 | 0.001739432 | 24.92 |
| class Verrucomicrobiae | rs12908520 | G | A | 0.062 | 0.013 | 0.433 | 2.17E-06 | 0.001559183 | 22.34 |
| class Verrucomicrobiae | rs2602429 | T | C | -0.075 | 0.016 | 0.780 | 2.58E-06 | 0.001595583 | 22.86 |
| class Verrucomicrobiae | rs4242783 | A | G | -0.069 | 0.015 | 0.739 | 2.64E-06 | 0.001520224 | 21.78 |
| class Verrucomicrobiae | rs4936098 | G | A | -0.065 | 0.014 | 0.625 | 1.12E-06 | 0.001590201 | 22.78 |
| class Verrucomicrobiae | rs61779207 | G | A | -0.076 | 0.017 | 0.182 | 6.72E-06 | 0.001426157 | 20.43 |
| class Verrucomicrobiae | rs74542928 | T | C | 0.112 | 0.024 | 0.099 | 1.63E-06 | 0.001570834 | 22.50 |
| class Verrucomicrobiae | rs9349825 | A | G | -0.070 | 0.015 | 0.266 | 2.54E-06 | 0.001598035 | 22.89 |
| class Verrucomicrobiae | rs941682 | G | A | -0.063 | 0.014 | 0.275 | 9.61E-06 | 0.001346594 | 19.29 |
| family Verrucomicrobiaceae | rs111862613 | T | C | 0.091 | 0.020 | 0.171 | 3.73E-06 | 0.001483549 | 21.25 |
| family Verrucomicrobiaceae | rs117107102 | A | G | 0.205 | 0.043 | 0.042 | 2.92E-06 | 0.001569844 | 22.49 |
| family Verrucomicrobiaceae | rs11729256 | T | C | 0.075 | 0.015 | 0.259 | 6.73E-07 | 0.001739432 | 24.92 |
| family Verrucomicrobiaceae | rs12908520 | G | A | 0.062 | 0.013 | 0.433 | 2.15E-06 | 0.001560079 | 22.35 |
| family Verrucomicrobiaceae | rs2602429 | T | C | -0.075 | 0.016 | 0.780 | 2.70E-06 | 0.001589902 | 22.78 |
| family Verrucomicrobiaceae | rs4242783 | A | G | -0.069 | 0.015 | 0.739 | 2.75E-06 | 0.001514484 | 21.70 |
| family Verrucomicrobiaceae | rs4936098 | G | A | -0.065 | 0.014 | 0.625 | 1.13E-06 | 0.001589457 | 22.77 |
| family Verrucomicrobiaceae | rs61779207 | G | A | -0.076 | 0.017 | 0.182 | 6.63E-06 | 0.001428043 | 20.46 |
| family Verrucomicrobiaceae | rs74542928 | T | C | 0.112 | 0.024 | 0.099 | 1.65E-06 | 0.001569715 | 22.49 |
| family Verrucomicrobiaceae | rs9349825 | A | G | -0.070 | 0.015 | 0.266 | 2.51E-06 | 0.001599477 | 22.92 |
| family Verrucomicrobiaceae | rs941682 | G | A | -0.063 | 0.014 | 0.275 | 9.58E-06 | 0.001346978 | 19.29 |
| genus Akkermansia | rs111862613 | T | C | 0.091 | 0.020 | 0.171 | 3.39E-06 | 0.001497052 | 21.45 |
| genus Akkermansia | rs117107102 | A | G | 0.204 | 0.043 | 0.042 | 3.01E-06 | 0.001565192 | 22.42 |
| genus Akkermansia | rs11729256 | T | C | 0.075 | 0.015 | 0.259 | 6.58E-07 | 0.001742397 | 24.97 |
| genus Akkermansia | rs12908520 | G | A | 0.062 | 0.013 | 0.433 | 2.26E-06 | 0.001552933 | 22.25 |
| genus Akkermansia | rs2602429 | T | C | -0.075 | 0.016 | 0.780 | 2.72E-06 | 0.001589082 | 22.77 |
| genus Akkermansia | rs4242783 | A | G | -0.069 | 0.015 | 0.739 | 3.00E-06 | 0.001503205 | 21.53 |
| genus Akkermansia | rs4936098 | G | A | -0.065 | 0.014 | 0.625 | 1.10E-06 | 0.001591931 | 22.81 |
| genus Akkermansia | rs61779207 | G | A | -0.076 | 0.017 | 0.182 | 6.32E-06 | 0.001434425 | 20.55 |
| genus Akkermansia | rs74542928 | T | C | 0.113 | 0.024 | 0.099 | 1.48E-06 | 0.001583536 | 22.69 |
| genus Akkermansia | rs9349825 | A | G | -0.070 | 0.015 | 0.266 | 2.60E-06 | 0.001595077 | 22.85 |
| genus Akkermansia | rs941682 | G | A | -0.063 | 0.014 | 0.275 | 9.17E-06 | 0.001352893 | 19.38 |
| genus Dialister | rs10138457 | T | C | -0.113 | 0.026 | 0.066 | 7.88E-06 | 0.001300493 | 18.63 |
| genus Dialister | rs10938938 | A | G | 0.077 | 0.017 | 0.809 | 7.37E-06 | 0.001429672 | 20.48 |
| genus Dialister | rs11071887 | T | C | 0.066 | 0.015 | 0.271 | 5.91E-06 | 0.001430629 | 20.49 |
| genus Dialister | rs11166701 | G | A | -0.066 | 0.013 | 0.509 | 5.51E-07 | 0.001723408 | 24.69 |
| genus Dialister | rs2314294 | T | C | 0.087 | 0.019 | 0.140 | 8.08E-06 | 0.001394758 | 19.98 |
| genus Dialister | rs2435610 | A | C | 0.065 | 0.014 | 0.284 | 5.93E-06 | 0.001423219 | 20.39 |
| genus Dialister | rs4747450 | C | A | 0.067 | 0.015 | 0.266 | 5.84E-06 | 0.001430217 | 20.49 |
| genus Dialister | rs4753063 | A | G | 0.060 | 0.013 | 0.551 | 4.86E-06 | 0.001467355 | 21.02 |
| genus Dialister | rs75416973 | A | G | 0.073 | 0.016 | 0.191 | 9.46E-06 | 0.001364079 | 19.54 |
| genus Dialister | rs764177 | C | A | -0.060 | 0.014 | 0.318 | 9.61E-06 | 0.001378061 | 19.74 |
| genus Dialister | rs76680460 | G | A | -0.161 | 0.036 | 0.055 | 8.19E-06 | 0.001368169 | 19.60 |
| genus Faecalibacterium | rs10927394 | G | T | -0.232 | 0.051 | 0.031 | 7.02E-06 | 0.001434358 | 20.55 |
| genus Faecalibacterium | rs114946999 | C | T | -0.086 | 0.019 | 0.096 | 5.70E-06 | 0.001441301 | 20.65 |
| genus Faecalibacterium | rs11776390 | T | C | -0.078 | 0.017 | 0.112 | 6.40E-06 | 0.001451361 | 20.79 |
| genus Faecalibacterium | rs1271565 | C | T | -0.058 | 0.012 | 0.272 | 1.30E-06 | 0.001618764 | 23.19 |
| genus Faecalibacterium | rs12753492 | A | C | 0.064 | 0.015 | 0.161 | 8.80E-06 | 0.001277208 | 18.29 |
| genus Faecalibacterium | rs2835874 | T | C | -0.087 | 0.020 | 0.073 | 7.54E-06 | 0.001356992 | 19.44 |
| genus Faecalibacterium | rs6910935 | G | A | -0.135 | 0.028 | 0.942 | 1.38E-06 | 0.001653846 | 23.70 |
| genus Faecalibacterium | rs75499067 | C | T | 0.228 | 0.047 | 0.052 | 1.76E-06 | 0.001667876 | 23.90 |
| genus Faecalibacterium | rs79656633 | T | C | 0.146 | 0.032 | 0.053 | 8.14E-06 | 0.001418785 | 20.32 |
| genus Faecalibacterium | rs9536330 | T | C | -0.048 | 0.011 | 0.393 | 5.33E-06 | 0.001397874 | 20.02 |
| genus Lachnospiraceae ND3007 group | rs2861203 | G | A | 0.057 | 0.013 | 0.296 | 7.37E-06 | 0.001411265 | 20.22 |
| genus Lachnospiraceae ND3007 group | rs72776675 | T | C | -0.065 | 0.015 | 0.182 | 8.72E-06 | 0.001334913 | 19.12 |
| genus Lachnospiraceae ND3007 group | rs9932954 | A | G | -0.056 | 0.012 | 0.456 | 1.25E-06 | 0.001637707 | 23.46 |
| genus Oscillibacter | rs11627628 | T | C | 0.144 | 0.029 | 0.078 | 1.01E-06 | 0.001716965 | 24.60 |
| genus Oscillibacter | rs11990279 | T | C | -0.082 | 0.018 | 0.240 | 4.94E-06 | 0.001458609 | 20.89 |
| genus Oscillibacter | rs12649930 | T | G | 0.122 | 0.026 | 0.087 | 4.09E-06 | 0.00153093 | 21.93 |
| genus Oscillibacter | rs133832 | A | C | -0.080 | 0.016 | 0.325 | 1.15E-06 | 0.001674299 | 23.99 |
| genus Oscillibacter | rs16866406 | A | G | 0.099 | 0.021 | 0.137 | 3.08E-06 | 0.00156512 | 22.42 |
| genus Oscillibacter | rs16934185 | A | G | -0.130 | 0.028 | 0.089 | 4.38E-06 | 0.001477958 | 21.17 |
| genus Oscillibacter | rs234108 | A | G | 0.075 | 0.015 | 0.479 | 9.16E-07 | 0.001682921 | 24.11 |
| genus Oscillibacter | rs36095275 | C | T | -0.075 | 0.016 | 0.375 | 1.40E-06 | 0.001605516 | 23.00 |
| genus Oscillibacter | rs4506202 | G | A | 0.071 | 0.015 | 0.464 | 3.21E-06 | 0.001523267 | 21.82 |
| genus Oscillibacter | rs61883564 | A | G | -0.101 | 0.022 | 0.135 | 3.39E-06 | 0.001467751 | 21.03 |
| genus Oscillibacter | rs62206502 | A | C | 0.068 | 0.015 | 0.544 | 6.60E-06 | 0.001414951 | 20.27 |
| genus Oscillibacter | rs75453768 | G | T | 0.122 | 0.027 | 0.093 | 5.35E-06 | 0.001442523 | 20.66 |
| genus Oscillibacter | rs761240 | G | T | 0.177 | 0.039 | 0.925 | 2.04E-06 | 0.001440637 | 20.64 |
| genus Oscillibacter | rs9393920 | G | A | 0.074 | 0.015 | 0.530 | 9.92E-07 | 0.001695288 | 24.29 |
| genus Peptococcus | rs10031059 | C | T | 0.121 | 0.023 | 0.768 | 1.24E-07 | 0.002007985 | 28.78 |
| genus Peptococcus | rs11001941 | G | A | -0.196 | 0.039 | 0.066 | 1.33E-06 | 0.001735646 | 24.87 |
| genus Peptococcus | rs12069354 | C | T | 0.168 | 0.038 | 0.064 | 9.28E-06 | 0.00136197 | 19.51 |
| genus Peptococcus | rs2054133 | A | G | -0.090 | 0.019 | 0.487 | 2.14E-06 | 0.001577662 | 22.60 |
| genus Peptococcus | rs36121075 | A | G | -0.141 | 0.031 | 0.112 | 6.99E-06 | 0.001472338 | 21.09 |
| genus Peptococcus | rs413827 | G | A | 0.110 | 0.024 | 0.206 | 3.30E-06 | 0.001503173 | 21.53 |
| genus Peptococcus | rs5770862 | T | C | 0.162 | 0.036 | 0.074 | 3.22E-06 | 0.001439135 | 20.62 |
| genus Peptococcus | rs6918730 | A | G | -0.135 | 0.029 | 0.886 | 1.15E-06 | 0.001522171 | 21.81 |
| genus Peptococcus | rs7033353 | G | T | -0.090 | 0.019 | 0.600 | 2.22E-06 | 0.001572066 | 22.52 |
| genus Peptococcus | rs72850165 | T | C | -0.134 | 0.030 | 0.112 | 5.74E-06 | 0.001395041 | 19.98 |
| genus Peptococcus | rs74592222 | G | A | 0.138 | 0.030 | 0.108 | 8.55E-06 | 0.001447289 | 20.73 |
| genus Peptococcus | rs77681628 | C | T | 0.200 | 0.039 | 0.064 | 2.69E-07 | 0.001865972 | 26.74 |
| genus Ruminococcaceae UCG003 | rs10490280 | C | T | -0.067 | 0.014 | 0.244 | 4.16E-06 | 0.001535068 | 21.99 |
| genus Ruminococcaceae UCG003 | rs11243416 | T | C | -0.093 | 0.019 | 0.101 | 1.67E-06 | 0.001635603 | 23.43 |
| genus Ruminococcaceae UCG003 | rs11613919 | G | T | 0.073 | 0.016 | 0.164 | 1.63E-06 | 0.001523853 | 21.83 |
| genus Ruminococcaceae UCG003 | rs16959793 | A | C | -0.063 | 0.013 | 0.275 | 2.22E-06 | 0.001583691 | 22.69 |
| genus Ruminococcaceae UCG003 | rs2523124 | C | T | 0.055 | 0.012 | 0.625 | 5.78E-06 | 0.001428699 | 20.47 |
| genus Ruminococcaceae UCG003 | rs3013089 | G | A | -0.055 | 0.012 | 0.404 | 4.38E-06 | 0.001465649 | 21.00 |
| genus Ruminococcaceae UCG003 | rs4452755 | A | C | -0.063 | 0.013 | 0.277 | 3.29E-06 | 0.00154747 | 22.17 |
| genus Ruminococcaceae UCG003 | rs4532474 | G | A | 0.077 | 0.017 | 0.130 | 4.82E-06 | 0.00142162 | 20.36 |
| genus Ruminococcaceae UCG003 | rs646327 | G | A | 0.059 | 0.012 | 0.528 | 7.83E-07 | 0.0017143 | 24.56 |
| genus Ruminococcaceae UCG003 | rs6759615 | A | G | 0.103 | 0.020 | 0.076 | 7.86E-07 | 0.001829715 | 26.22 |
| genus Ruminococcaceae UCG003 | rs73341549 | T | C | -0.170 | 0.032 | 0.049 | 1.51E-07 | 0.001980789 | 28.39 |
| genus Ruminococcaceae UCG003 | rs78720113 | A | G | -0.115 | 0.025 | 0.072 | 7.59E-06 | 0.001490601 | 21.35 |
| genus Streptococcus | rs10028567 | C | T | -0.092 | 0.019 | 0.092 | 7.30E-06 | 0.001608405 | 23.04 |
| genus Streptococcus | rs10448310 | A | G | -0.052 | 0.011 | 0.435 | 3.31E-06 | 0.001510769 | 21.64 |
| genus Streptococcus | rs11110281 | T | C | -0.138 | 0.023 | 0.063 | 2.58E-09 | 0.002549911 | 36.57 |
| genus Streptococcus | rs11720390 | G | A | 0.107 | 0.023 | 0.079 | 3.59E-06 | 0.001536194 | 22.01 |
| genus Streptococcus | rs11764382 | A | G | -0.070 | 0.014 | 0.182 | 1.29E-06 | 0.001634684 | 23.42 |
| genus Streptococcus | rs17708276 | A | G | -0.079 | 0.017 | 0.138 | 3.04E-06 | 0.00151118 | 21.65 |
| genus Streptococcus | rs1918540 | A | G | -0.060 | 0.013 | 0.738 | 2.44E-06 | 0.001511687 | 21.66 |
| genus Streptococcus | rs2370083 | G | T | -0.082 | 0.019 | 0.105 | 9.75E-06 | 0.001348453 | 19.31 |
| genus Streptococcus | rs4968759 | A | G | -0.052 | 0.011 | 0.430 | 3.78E-06 | 0.001474606 | 21.12 |
| genus Streptococcus | rs57646748 | G | A | -0.091 | 0.020 | 0.072 | 5.48E-06 | 0.001432805 | 20.52 |
| genus Streptococcus | rs6806351 | T | C | -0.063 | 0.014 | 0.233 | 4.94E-06 | 0.001501665 | 21.51 |
| genus Streptococcus | rs71481756 | T | G | 0.093 | 0.021 | 0.090 | 6.51E-06 | 0.001399278 | 20.04 |
| genus Streptococcus | rs72739637 | A | G | 0.096 | 0.019 | 0.113 | 1.03E-06 | 0.001722465 | 24.68 |
| genus Streptococcus | rs7916711 | A | G | 0.103 | 0.022 | 0.058 | 2.72E-06 | 0.00156383 | 22.40 |
| genus Streptococcus | rs9903102 | C | A | -0.071 | 0.016 | 0.141 | 4.18E-06 | 0.001457234 | 20.87 |
| order Verrucomicrobiales | rs111862613 | T | C | 0.091 | 0.020 | 0.171 | 3.74E-06 | 0.001483304 | 21.25 |
| order Verrucomicrobiales | rs117107102 | A | G | 0.205 | 0.043 | 0.042 | 2.92E-06 | 0.001569844 | 22.49 |
| order Verrucomicrobiales | rs11729256 | T | C | 0.075 | 0.015 | 0.259 | 6.73E-07 | 0.001739432 | 24.92 |
| order Verrucomicrobiales | rs12908520 | G | A | 0.062 | 0.013 | 0.433 | 2.17E-06 | 0.001559183 | 22.34 |
| order Verrucomicrobiales | rs2602429 | T | C | -0.075 | 0.016 | 0.780 | 2.58E-06 | 0.001595583 | 22.86 |
| order Verrucomicrobiales | rs4242783 | A | G | -0.069 | 0.015 | 0.739 | 2.64E-06 | 0.001520224 | 21.78 |
| order Verrucomicrobiales | rs4936098 | G | A | -0.065 | 0.014 | 0.625 | 1.12E-06 | 0.001590201 | 22.78 |
| order Verrucomicrobiales | rs61779207 | G | A | -0.076 | 0.017 | 0.182 | 6.72E-06 | 0.001426157 | 20.43 |
| order Verrucomicrobiales | rs74542928 | T | C | 0.112 | 0.024 | 0.099 | 1.63E-06 | 0.001570834 | 22.50 |
| order Verrucomicrobiales | rs9349825 | A | G | -0.070 | 0.015 | 0.266 | 2.54E-06 | 0.001598035 | 22.89 |
| order Verrucomicrobiales | rs941682 | G | A | -0.063 | 0.014 | 0.275 | 9.61E-06 | 0.001346594 | 19.29 |
| order Victivallales | rs1002941 | A | G | -0.105 | 0.023 | 0.726 | 8.15E-06 | 0.001412339 | 20.23 |
| order Victivallales | rs11770843 | C | T | 0.109 | 0.023 | 0.291 | 1.91E-06 | 0.001515011 | 21.70 |
| order Victivallales | rs17114848 | G | A | 0.152 | 0.032 | 0.123 | 4.06E-06 | 0.001540535 | 22.07 |
| order Victivallales | rs2031282 | A | G | 0.122 | 0.027 | 0.171 | 4.38E-06 | 0.001430246 | 20.49 |
| order Victivallales | rs2825714 | A | G | -0.137 | 0.029 | 0.147 | 1.72E-06 | 0.001575067 | 22.57 |
| order Victivallales | rs62570196 | C | T | -0.216 | 0.044 | 0.080 | 1.08E-06 | 0.001688189 | 24.19 |
| order Victivallales | rs72640280 | A | G | 0.220 | 0.049 | 0.058 | 5.18E-06 | 0.001431855 | 20.51 |
| order Victivallales | rs77599476 | A | G | 0.230 | 0.048 | 0.055 | 1.86E-06 | 0.001605297 | 23.00 |

A1: gut microbiome increasing allele; A2: other allele; SE: standard error; EAF: effect allele frequency
