# Supplementary material for: Causal relationship between the gut microbiome and basal cell carcinoma, melanoma skin cancer, ease of skin tanning: evidence from three two-sample mendelian randomisation studies
Source: Front Immunol. 2024 Jan 18;15:1279680. doi: 10.3389/fimmu.2024.1279680 (PMC10830803; doi:10.3389/fimmu.2024.1279680)
Supplement: Supplementary file 16 [file Table_4.docx]

**Supplementary Table 4. Sensitivity and pleiotropy tests for gut microbiota and basal cell carcinoma, melanoma skin cancer and ease of skin tanning.**

| Exposure | Heterogeneity test | | | | |  | Pleiotropy test | | |
| --- | --- | --- | --- | --- | --- | --- | --- | --- | --- |
|  | IVW | | MR-Egger | | |  | MR-Egger | | |
|  | Cochran's Q | Q_pval | Cochran's Q | Q_pval |  | | Intercept | SE | p-value |
| **Basal cell carcinoma** |  |  |  |  |  | |  |  |  |
| family Family XI | 4.583 | 0.711 | 4.516 | 0.607 |  | | 0.0071 | 0.027 | 0.805 |
| genus Clostridium innocuum group | 4.4 | 0.623 | 1.276 | 0.937 |  | | -0.047 | 0.027 | 0.137 |
| genus Family XIII AD3011 group | 10.94 | 0.448 | 10.86 | 0.368 |  | | -0.0067 | 0.025 | 0.796 |
| genus Parabacteroides | 3.276 | 0.658 | 2.232 | 0.693 |  | | -0.024 | 0.023 | 0.365 |
| genus Romboutsia | 8.036 | 0.782 | 7.213 | 0.782 |  | | 0.0098 | 0.011 | 0.384 |
| genus Ruminiclostridium5 | 4.978 | 0.836 | 4.633 | 0.796 |  | | -0.011 | 0.019 | 0.573 |
| genus Ruminococcaceae UCG014 | 3.509 | 0.941 | 3.507 | 0.899 |  | | 0.00062 | 0.012 | 0.961 |
| genus Turicibacter | 8.698 | 0.466 | 7.206 | 0.515 |  | | 0.026 | 0.022 | 0.257 |
| **Melanoma skin cancer** |  |  |  |  |  | |  |  |  |
| genus Parabacteroides | 2.905 | 0.715 | 2.905 | 0.574 |  | | 7.1e-06 | 5e-04 | 0.989 |
| genus Prevotella7 | 9.935 | 0.356 | 9.903 | 0.272 |  | | -9.5e-05 | 0.00059 | 0.876 |
| genus Ruminococcaceae UCG013 | 13.22 | 0.279 | 12.7 | 0.241 |  | | 0.00017 | 0.00027 | 0.536 |
| genus Veillonella | 3.495 | 0.745 | 1.011 | 0.962 |  | | 0.00071 | 0.00045 | 0.176 |
| **Ease of skin tanning** |  |  |  |  |  | |  |  |  |
| class Lentisphaeria | 4.667 | 0.701 | 4.410 | 0.621 |  | | 0.0018 | 0.0036 | 0.631 |
| class Verrucomicrobiae | 71.090 | 2.725E-11 | 66.130 | 8.688E-11 |  | | 0.0058 | 0.007 | 0.432 |
| family Verrucomicrobiaceae | 71.070 | 2.755E-11 | 66.140 | 8.651E-11 |  | | 0.0058 | 0.007 | 0.434 |
| genus Akkermansia | 71.050 | 2.78E-11 | 66.150 | 8.612E-11 |  | | 0.0057 | 0.007 | 0.435 |
| genus Dialister | 4.535 | 0.920 | 4.535 | 0.873 |  | | 0.000021 | 0.003 | 0.995 |
| genus Faecalibacterium | 10.940 | 0.280 | 10.160 | 0.254 |  | | -0.0018 | 0.0022 | 0.456 |
| genus Lachnospiraceae ND3007 group | 0.255 | 0.880 | 0.255 | 0.613 |  | | -0.000005 | 0.022 | 1.00 |
| genus Oscillibacter | 11.340 | 0.583 | 9.208 | 0.685 |  | | -0.0036 | 0.0024 | 0.170 |
| genus Peptococcus | 16.840 | 0.113 | 16.770 | 0.080 |  | | 0.0046 | 0.0025 | 0.095 |
| genus Ruminococcaceae UCG003 | 12.720 | 0.312 | 9.322 | 0.502 |  | | -0.0012 | 0.0032 | 0.719 |
| genus Streptococcus | 18.260 | 0.195 | 18.070 | 0.155 |  | | 0.0058 | 0.007 | 0.432 |
| order Verrucomicrobiales | 71.090 | 2.725E-11 | 66.130 | 8.688E-11 |  | | 0.0018 | 0.0036 | 0.631 |
| order Victivallales | 4.667 | 0.701 | 4.410 | 0.621 |  | | -0.00091 | 0.0043 | 0.835 |
